# Cohort profile: The Belgian I AM frontier prospective cohort study for comprehensive health outcome exploration

**DOI:** 10.1371/journal.pone.0326024

**Published:** 2025-06-12

**Authors:** Dries Heylen, Charlotte De Clerck, Murih Pusparum, Alejandro Correa Rojo, Rosette Van Den Heuvel, Geert Baggerman, Arnout Standaert, Jan Theunis, Jef Hooyberghs, Gökhan Ertaylan, Nathalie Lambrechts

**Affiliations:** 1 Environmental intelligence Unit, Flemish Institute for Technological Research (VITO), Mol, Belgium; 2 Theory lab, Data Science Institute (DSI), CENSTAT, Hasselt University, Diepenbeek, Belgium; 3 Data Science Institute (DSI), CENSTAT, Hasselt University, Diepenbeek, Belgium; 4 Center for Proteomics, University of Antwerp, Antwerp, Belgium; Karolinska Institutet, SWEDEN

## Abstract

**Objectives:**

The I AM Frontier cohort was set up to support proof-of-concepts aimed at personalized prevention and health promotion. The study was designed to identify patterns, markers and processes involved in the spectrum between health and early disease onset, with the aim of generating actionable insights in a clinical setting.

**Study design:**

A prospective cohort study. The study was approved by the ethics committee of the Antwerp University Hospital (RegN°: B300201938600).

**Methods:**

Data collection in the I AM Frontier study spanned 12 months as a longitudinal small-scale cohort study (n = 30) conducted in the Antwerp region of Flanders, Belgium. Participants were employees of the company hosting the study, who did not have a clinical diagnosis and were between 45–60 years old.

**Results:**

Even though no severe health problems were recorded at baseline, participants reported several physical complaints. There was a clear difference in longitudinal variation between clinical and research grade omics types, which might have affected their respective ability to detect intermediate molecular changes that were be linked to phenotype changes.

**Conclusion:**

By integrating findings from the IAF cohort into a large-scale cohort on personalized prevention, and incorporating new technologies for microsample data collection and participant engagement, we can develop more precise and individualized health recommendations.

## Introduction

The healthcare landscape is evolving, and several trends push the current socio-technical regime beyond its (financial) capacity. With an aging population, the prevalence of chronic diseases such as diabetes, cardiovascular diseases, and cancer is increasing, along with disease co-morbidity. The European Union Policy Forum estimates that about 70–80% of the total healthcare cost in the EU (about 700 billion Euros) is earmarked for chronic care. Furthermore, the WHO predicts that partially preventable diseases will constitute the main contribution to the burden of disease in 2030. The indirect costs, including loss of income, inability to work, and informal care, are a substantial additional cost to society [1,2].

On top of the increasing financial and social costs, there is growing scientific consensus that conventional approaches towards disease and therapy are not generating sufficient new solutions for complex diseases, and that health promotion and data-driven disease prevention are the way forward [3,4]. Therefore, a reshaping of the socio-technical regime is imperative. Society must evolve towards a more sustainable healthcare system. In that light, the concept of ‘precision health’ is introduced, focusing on a holistic approach to healthcare, maintaining and improving health by encompassing a wide range of factors influencing health, such as behavior and environmental factors. As science understands more about a person’s biological makeup, it becomes clear that many causes of disease and causes of unresponsiveness or serious side effects to treatments can be explained by the variability in an individual’s characteristics. Precision health tackles these problems by moving away from a ‘one size fits all’ approach by integrating data on the dynamic biological makeup of individuals, and the lifestyle and environmental factors that interact with this makeup, to develop a complex and individual phenotype [5,6]. So-called ‘personalized prevention,’ on the other hand, is a specific aspect of precision health that envisions a personal approach for disease prevention and health promotion [7].

Recent technological advancements, such as personal sensor technologies, and developments in various ‘omics’ fields, such as genomics, proteomics, and transcriptomics, increase the potential of precision health by improving the comprehension of an individual’s physiological state. Understanding the integrations of underlying biological processes involved in disease onset and development, rather than looking at isolated parameters of these biological processes, provides opportunities for increased prevention, better diagnostics and ultimately more efficient treatment. The integrated Personal Omic Profiling (iPOP) system by Michael Snyder in 2012 [8] is an analysis methodology that combines multi-omics information with monitoring of physiological state. IPOP tracks a wide range of biological data over time, creating a dynamic picture of an individual’s health. Studies following this nascent effort have demonstrated that longitudinal iPOP can be used to interpret subtle changes in healthy and diseased states [9,10].

### Objectives and aims of the I AM frontier study

Today, various biobanks and cohorts collect samples and perform in depth phenotyping of individuals with the purpose of facilitating precision health across various biomedical domains [9,11–15]. In that light the term ‘omics’ is often used to represent a complete set of molecules within a biological system. However, data collection in a cohort with healthy individuals by integrating extensive omics, clinical and continuous personal monitoring data, is still scarce. This type of cohort study aims to provide insights into the earliest manifestation of chronic diseases and enable tracking disease progression. The I AM frontier proof-of-concept (POC) study was set up as a first step towards larger scale-ups by gaining specific knowledge on the added clinical value of high frequent tracking of a generally healthy cohort of individuals with clinical tests, omics tests, questionnaires, and wearables while considering ethical and legal aspects. The study investigates candidate processes (or molecules) tightly regulated in health (considering seasonality) and their deviation from their normal pattern, as this could signal the onset of a spectrum of diseases. Besides the clinical aspects, we’ve also considered the ethical and societal impact by studying best practice on how to communicate gained insights to individuals informing them about potential health threats whilst avoiding ungrounded life-disrupting messages.

## Materials and methods

### Cohort description

**Fig 1:**
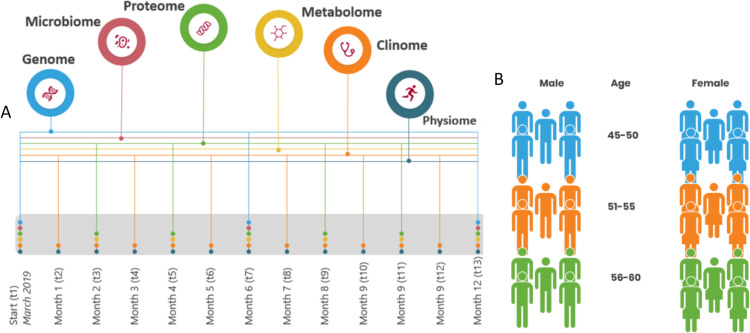
A) Data collection flow diagram. Datatypes were collected at different time points, circle stacks on each time point indicate which datatypes were collected at that moment in time (n). B) Gender and age distribution of the cohort. Cohort consisted of 30 participants (n = 30).

The I AM Frontier study ran for 12 months, starting in March 2019 and ending in March 2020, as a proof-of-concept cohort study aimed at advancing personalized disease prevention (n = 30) in the Antwerp region of Flanders, Belgium (Fig 1). The cohort included comprehensive longitudinal multi-omics, clinical, and sensor-based health monitoring data. This, together with wide-ranging questionnaires, created detailed phenotyping of healthy individuals.

Besides the scientific results, also logistical and ethical know-how was obtained with the cohort. The logistic results focussed on how the study collected, stored, and analyzed various samples and data types (blood, urine, hair, saliva, stool, retina scans), either monthly or (for some) quarterly. Various steps in the processes had different sensitivities, processing times, and operating costs. Logistical processes and workflows were created and reported to enable future optimization efforts when scaling up similar cohort studies. Ethical insights were gained by interviewing participants regarding their perspective on personal health data and communication on its insights.

### Patient and public involvement: Study design and eligibility criteria

The IAM frontier cohort study exclusively targeted its own Flemish research institute’s employees, with the expectation that, due to the nature of their employer, employees would be more open to research-grade technologies. With its intensive protocol, the nature of the POC cohort required sufficient commitment and understanding of what was asked and of the risks and uncertainty of what may be found. Unlike representativeness, which was not a requisite in this POC study, feasibility was a key consideration. Other reasons for this target population were also of a practical nature, i.e., the occupational health service did sample collection during working hours. Privacy and avoidance of any pressure to participate or remain in the study were given the highest priority.

**Fig 2:**
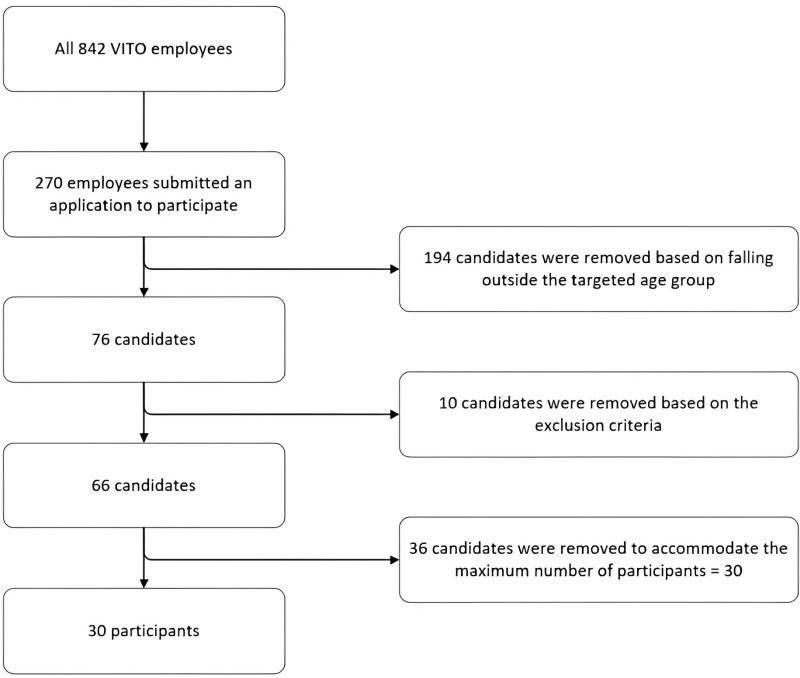
Candidate selection process.

All 842 VITO employees were initially approached through a company-wide information session and the internal website. Subsequently, from the 270 interested candidates, 76 met the age requirement: an age range of 45−65 was chosen because it encompasses the period during which chronic diseases, such as diabetes, COPD, heart diseases, and athritis, are most commonly initiated [16]. These 76 candidates were contacted for an interview and received additional general information about the project. A set of exclusion criteria was used, rejecting people suffering from a chronic disease (i.e., diagnosed and currently followed-up by a medical specialist for asthma, chronic bronchitis, chronic obstructive pulmonary disease, emphysema, myocardial infarction, coronary heart disease (angina pectoris), other serious heart disease, stroke (cerebral hemorrhage, cerebral thrombosis), diabetes and cancer (malignant tumor, also including leukemia and lymphoma)). The researchers ensured that none of them were frequent blood-donors (due to interference with sampling protocol), were active smartphone users (as some degree of digital literacy was required throughout the study), and that their motivation was not based on personal health gain but research-oriented. Based on these criteria, 66 of the 76 candidates were considered eligible. Next, we ranked them according to age and selected 30 participants with an equally spread age distribution in the 45−60 age range, and an equal amount of female and male participants (Fig 1B). The candidate selection process is shown in a flow diagram in Fig 2. All 30 participants remained in the study throughout the first year of the sampling process. Only in the very last month of the sampling procedure we experienced ten dropouts due to the COVID-19 pandemic and lock-down that was in place during sampling. All selected participants signed an informed consent prior to enrollment, at the time they were informed of their selection. The study was approved by the ethics committee of the Antwerp University Hospital (RegN°: B300201938600).

### Patient and public involvement: Feedback to the participants

Participants received three distinct types of results: health prevention actionable items, genetic results, and biomarkers for scientific use. The first group consisted of clinical parameters associated with a known adverse outcome. Participant received their personal clinical parameter values compared to normal clinical ranges, provided by AML Flanders [17], through individual reports on a bi-monthly basis. The study physician reviewed these reports with participants every four months and provided guidance on interventions that could positively influence these parameters. The second group of results encompassed so-called ‘incidental findings’ (IF): genetic alterations associated with conditions or diseases unrelated to the patient’s or participant’s current condition for which current tests are being performed. For feedback on IF or discovery findings based on IF the ‘ACMG Recommendations for Reporting of Incidental Findings in Clinical Exome and Genome Sequencing’ [18,19] were followed. The third group contained all other ‘omics’ parameters associated with insufficient evidence-base to an adverse outcome in scientific literature to be communicated to the study physician or participants. These results were strictly used for research purposes only.

### Study sampling and data types

The sample collection period took place between March 2019 and March 2020 and included the collection of various human biospecimens (blood, urine, saliva, stool, and hair) and monitoring data (wearables, questionnaires, and retina scans). See Table 1 for an overview of all collected data, the collection time frames, the measurement techniques, and the specific laboratories in which the different analyses were performed within the I AM Frontier POC study.

**Table 1:** Visual of the data collection for I AM Frontier Cohort. A parameter specific overview of the measured variables for metabolomics, proteomics and clinical parameters can be found in supplementary tables.

Data	Number of parameters per sampling point	Measurement technique (and the laboratory executing)	Timepoints – month	Type of sample
**Metabolomics (Metabolome)**	220 NMR & 190 MS parameters	NMR – MS (Nightingale health)	0-2-4-6-8-10-12	Plasma
**Proteomics (Proteome)**	1068 distinct parameters	LC-MSMS -PEA (OLINK)	0-2-4-6-8-10-12	Plasma
**Clinical parameters (clinome)**	Variety of measurement techniques applied by AML Labs		0-1-2-3-4-5-6-7-8-9-10-11-12	Whole blood
	108 parameters	Clinical biochemistry, blood cell counts	0-1-2-3-4-5-6-7-8-9-10-11-12	Whole blood**,** serum PB**,** fasting urine
	18 parameters	Immune cells, vitamins	0-2-4-6-8-10-12	Whole blood**,** serum PB
	104 parameters	All other clinical tests	Up to 3 time points, irregular	Whole blood, serum PB, fasting urine
**Full genome sequencing**	Whole genome	BGISEQ Human Whole Genome Sequencing for 30X protocol (BGI Genomics)	1	Saliva
**Epigenetics**	Information for > 850 000 CpGs	Illumina SCAN-data	0-5-12	PBMC
**Imaging, sensors and wearables**	>200 parameters	Range of digital devices and retina scans	0-1-2-3-4-5-6-7-8-9-10-11-12	Mixed
**Microbiome**	Atlas biomed commercial kit	Illumina miseq (Atlas biomed)	0-5-12	Stool
**Questionnaires:**	Different types on different timepoints	Questions asked digitally	0-5	Questionnaires
** Intake**	54 questions	General health condition	0	Questionnaires
** Monthly**	59 questions	Physical activity and food intake		Questionnaires
** Weekly**	33 questions	Health condition and symptoms		Questionnaires
** WGS perception**	20 questions	Whole genome sequencing perception	Pre and post	Questionnaires
**Biobanking**	All above collected biospecimen samples	Laboratory information management system (LIMS)	0-1-2-3-4-5-6-7-8-9-10-11-12	Blood, plasma, urine, stool, saliva & hair
** Registration**	All collected biospecimen samples	Laboratory information management system (LIMS)	0-1-2-3-4-5-6-7-8-9-10-11-12	Blood, plasma, serum, urine, PBMC, stool, saliva & hair
** Long-term storage**	All collected biospecimen	Laboratory information	0-1-2-3-4-5-6-7-8-9-10-11-12	Plasma, urine, Peripheral blood mononuclear cells (PBMNC)

### Digital monitoring

For digital monitoring data, all participants received a Garmin Vivosmart 4 activity watch to record their heart rate, activity, and sleep on a continuous basis, ensuring accurate readings. We collected several physiological parameters—including steps, distance, speed, calories burned, heart rate, and activity duration—specifically during periods of physical activity such as walking, running, cycling, or sleeping. The watch was typically charged for approximately one to two hours each day, during which time it was not worn and thus did not record data. To facilitate analysis, all recorded data were averaged per calendar day.

Retinal imaging was performed at the sample collection visits using a Canon CR-2 non-mydriatic digital retinal camera with a CE mark in accordance with European regulations (Hospithera, Brussels, Belgium). The method of De Boever and colleagues was used [20,21].

### Questionnaire monitoring

Questionnaire monitoring data were gathered through self-administered questionnaires completed by participants to assess their personal lifestyle, environment, and health status. At the start of the study, a questionnaire was used to formalize the participant’s intake status, including information on lifestyle, environment, and previous or ongoing diseases. Their responses were considered baseline results.

Each month, the day before biospecimen sampling, participants completed questionnaires on daily activity and lifestyle patterns (nutrition, smoking, etc.). Additionally, a weekly questionnaire, based on the Cohen-Hoberman Inventory of Physical Symptoms (CHIPS) method, was used to assess their health and well-being over the past seven days [22]. This methodology consisted of 33 questions, asking participants to rate how much specific disorders -including infections, injuries, aches, fever, appetite, and weight changes- had bothered them during the previous week, on a scale from 0 (painless) to 4 (severe pain). Finally, in the WGS perception questionnaire, participants were asked about their perspectives on the ethical communication of insights derived from the genomics analyses, as well as their overall feedback on study participation.

All questionnaires (intake, monthly, weekly, and WGS perception) were sent and completed digitally. The full list of these questions can be consulted in S1 and S2 Files. The questions and respective answers were coded using a codebook. The coded data were stored directly on a data platform, accessible only to the involved researchers.

### Biological sampling

Participants were subjected to a monthly bio-sample collection. Sampling was done by a nurse and under supervision of an occupational physician at the in-house medical department. Blood pressure, body height, weight, and abdominal circumference were measured in a standardized way by the physician.

Blood was drawn by a nurse in the morning after a minimum of 8 hours of fasting. Multiple blood samples were collected in EDTA-, citrate- and serum- vacutainers (Greiner Bio-One International, Vilvoorde, Belgium). Blood was stored at room temperature till further processing. Serum was collected by centrifugation at 3500 rpm for 10 min. EDTA-whole blood, citrate whole blood, and serum were transported (at room temperature) to the clinical laboratory within 6 hours after collection for further processing and analysis. Plasma was removed by centrifugation at 3500 rpm for 5 min. Aliquots were made in pre-cooled tubes and immediately stored at −80°C. Leucosep^TM^ tubes (Greiner Bio-One International, Vilvoorde, Belgium) were used to isolate peripheral blood mononuclear cells (PBMC) from EDTA blood (30 min, 400xg). The PBMC suspension was divided into two aliquots (each originating from 5 mL of the original whole blood sample) and spun down (10 min, 250xg) to create PBMC pellets, which were stored at −80°C until the DNA extraction. Genomic DNA (gDNA) was extracted from PBMC pellets using the QIAamp DNA Blood Mini Kit (Qiagen, Antwerp, Belgium) according to the manufacturer’s protocol.

Urine samples were taken after an overnight fast and stored immediately at 4°C. Aliquots were stored at −80°C. Saliva samples were collected using Oragene OG-500 self-sampling kits (DNA Genotek, Ottawa, Canada). Stool samples were collected at home using Atlas Microbiome Tests (Atlas Biomed, London, UK). Saliva and stool samples were stored at room temperature till shipment for further analysis. A hairlock with a thickness of a match (2 mm) and a length of 4 cm was taken and stored in an envelope at room temperature.

### Biobank procedures

All biospecimen samples were registered at the Biobank@VITO (Mol, Belgium, ID:BB190064) and either stored temporarily in VITO’s biobank facilities until transfer to laboratories for analysis or stored long-term in VITO’s biobank facilities. Standard operating procedures (SOPs) to collect and process blood, urine, saliva, stool and hair samples were implemented. All samples were collected in accordance with the applicable Belgian regulations regarding the use of human body material for scientific research (Belgian Law on use of human body material, 2008) and the Belgian Royal Decree on biobanks (Het Koninklijk Besluit betreffende de biobanken. Belgisch Staatsblad 05.02.2018. Brussels [23]).

### Ethics approval and consent to participate

All participants signed an informed consent and the study was approved by the ethics committee of the Antwerp University Hospital (RegN°: B300201938600).

### Data analysis techniques

To correct for non-biological variance within the data, systematic data pre-processing was performed as prescribed by the laboratories performing the data measurement. An initial analysis of the data obtained from the diverse sampling technologies mentioned above was conducted to formulate the information presented in the ‘Findings to Data’ section. The study of Allen et al. [22] used principal component analysis with Varimax rotation and Kaiser Normalization to demonstrate that the 33 CHIPS questions concerning health complaints can be grouped into eight clusters. The identified physical health clusters were Sympathetic/Cardiac symptoms, Muscular pain, Metabolic symptoms, Gastrointestinal symptoms, Vasovagal symptoms, Cold/flu, and Minor haemorrhagic symptoms. All clusters except for ‘Minor haemorrhagic symptoms’ showed sufficient internal consistency in the study by Allen et al. [22].

The clinical results are summarized in some key clinical parameters, such as body mass index (BMI), Framingham risk score for cardiovascular disease [24], and the ADA diabetes risk score [25]. Clinical parameters were measured either each month, every other month, or only three times during the 12-month period. Each of the monthly clinical parameters is grouped in clinical health clusters: Adrenal, Allergy, Anemia, Carbohydrate metabolism, Cardiovascular, Hormonal, Immunological, Inflammation, Ionogram, Kidney, Liver, Proteins, and Thrombosis. The clinical health clusters were determined in consultation with the study physician, grouping together clinical parameters to simplify communication with participants. Measurements of the clinical parameters are compared to the normal interval for this parameter based on AML medical laboratory reference values [17,26]. Principal Component Analysis (PCA) was applied to perform dimensionality reduction of various types of omics data to explore potential clustering within our patient population. T-tests were used to compare clinical variables across different patient groups, assessing statistical differences between them. Population Reference Intervals (PRI) were consulted to evaluate whether individual clinical parameters fell within or outside normal population ranges, providing a framework for assessing deviations in patient data.

## Results

As this was the first phase of the I AM Cohort POC, we report the main the clinical, genetic, omics, and questionnaire findings.

### Questionnaire results

In the cohort, physical symptoms were self-reported with the CHIPS methodology. S1 Fig shows that at the start of the cohort study, more than 50% of the participants had sleeping problems and back pain. In total, 20% of the cohort reported their sleeping problems as serious. In addition, more than 30% of the participants suffered from constant fatigue, tense muscles, headache, muscle pain, and low energy at the start of the study.

**Fig 3:**
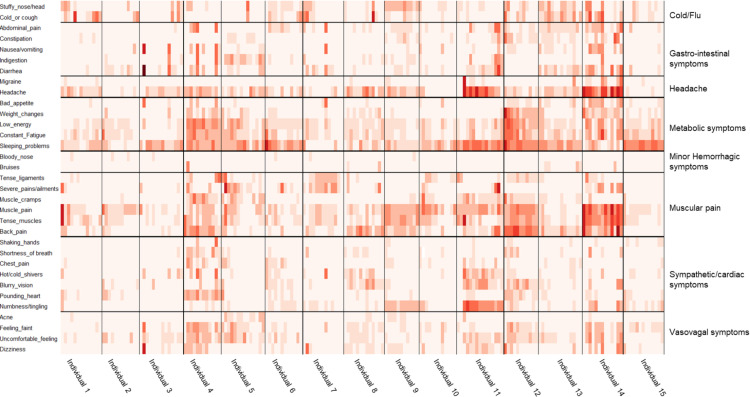
Physical symptoms throughout the study period (timepoints 0 to 12) were obtained via questionnaires and grouped according to the findings of Allen et. al [22]. The color intensity indicates the severity of the complaint on a scale from zero to four. Month zero is March 2019, and month 12 is March 2020. All time points are shown for the male participants. All time points of the female participants can be seen in S2 Fig.

To highlight the longitudinal aspect of physical symptoms in the cohort, Fig 3 shows an overview of the self-reported physical symptoms throughout the first phase sampling. The color indicates the severity of the complaint-the more intense the color the more a participant felt its impact. Each question falls within one of eight physical health clusters, indicated on the right of the figure [22]. An interesting finding from Fig 3 is that the metabolic symptoms cluster has the highest prevalence of reported symptoms, with sleep problems being one of the most frequently reported complaints. Additionally, it might be interesting to explore the higher prevalence of self-reported headaches in the female population compared to the male population. Over the longitudinal course of the study, most physical health clusters showed high internal consistency, as participants reported similar levels of severity for symptoms within one cluster. For certain specific symptoms, participants did experience fluctuations in severity. This was the case for indigestion, muscle pain, headache, sleeping problems, and back pain. Finally, symptoms of the Cold/Flu cluster were reported less during the summer months, as expected, with the Cold/Flu season situated during the winter months (Fig 3).

### Clinical results

Similar to the questionnaire-reported physical symptoms findings, a similar overview can be provided for the clinical measurement findings. At the start of the study, the body mass index (BMI) ranged from 19 to 34 kg/m2, with a mean of 25 kg/m2. On average, the BMI for men was significantly higher than for women. Also, the average blood pressure was lower in women (120/80 mmHg) than in men (129/84 mmHg). Only one person was identified as a current smoker, while the percentage of former smokers was lower among women (13%) than in men (27%). On average, the Framingham risk score for cardiovascular disease was 0.6 for women and 5.9 for men, corresponding to a 0.6% and 5.9% 10-year risk of a first ‘hard’ cardiovascular event (e.g., heart attack), respectively. The mean of the ADA diabetes risk score was below 5% in both women and men, corresponding to a low risk of type II diabetes mellitus. The detailed descriptive statistics of these parameters and the other clinical biochemistry parameters, such as total cholesterol, HDL cholesterol, and glucose, are reported in Table 2.

**Table 2:** Baseline characteristics at the time of recruitment of IAM Frontier by gender. The Student’s t-test was performed to determine if the means of each characteristic significantly differ between men and women.

Characteristic			Men	Women	P-value
Number of participants		Number	15	15	
Smoking status	Current	n (%)	1 (0.07)	0 (0.00)	
	Former	n (%)	4 (0.27)	2 (0.13)	
	Never	n (%)	10 (0.66)	13 (0.87)	
Age		Mean (SD)	50.8 (4.28)	50.7 (4.24)	0.9323
**BMI**		**Mean (SD)**	**27.3 (2.55)**	**23.0 (2.31)**	**<.0001**
Total cholesterol (mg/dL)		Mean (SD)	211 (35.6)	202 (36.2)	0.4915
**HDL cholesterol (mg/dL)**		**Mean (SD)**	**47.5 (10.6)**	**70.3 (10.1)**	**<.0001**
**Glucose level (mg/dL)**		**Mean (SD)**	**98.9 (5.77)**	**89.9 (8.95)**	**0.0032**
Systolic blood pressure (mmHg)		Mean (SD)	129.1 (13.0)	120.0 (11.2)	0.0502
Diastolic blood pressure (mmHg)		Mean (SD)	84.1 (9.3)	80.3 (7.3)	0.2214
**CVD risk score**		**Mean (SD)**	**5.9 (3.2)**	**0.6 (0.5)**	**<.0001**
**ADA Diabetes risk score** *		**Mean (SD)**	**2.0 (1.0)**	**4.1 (1.2)**	**<.0001**

**Fig 4:**
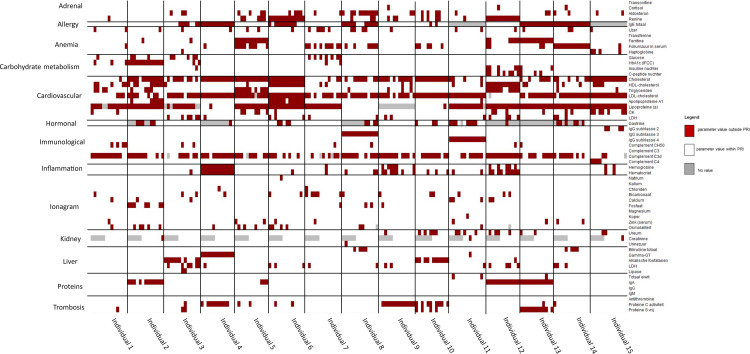
Longitudinal heatmap representing which clinical parameters (right), grouped in clinical health clusters (left), are out of normal range bounds, i.e., population reference intervals (PRI), for male participants. The heatmap with female participants can be found in S3 Fig.

Fig 4 is a visual representation of the monthly measured clinical parameters for each participant during the one-year course of the study, grouped into clinical health clusters, indicated on the right axis of the figure. A red measurement indicates the clinical parameter was out of bounds compared to the normal interval for this parameter. If the value was within normal bounds, it is represented in white. Values represented in grey indicate a missing value. Fig 4 shows clinical parameters for the male population, while S3 Fig shows those for the female population.

All male and female participants had at least one clinical parameter that was out of bounds at some time. A limited number of participants showed an obvious change in the clinical parameters over time. One of the most striking patterns was the C3d complement parameter, which was out of bounds (increased) for every participant, at least at some point during the sampling period. High C3d complement levels point towards an increased activation of the immune response through activation of the complement system [27]. Besides that, we observed an obvious difference in the cardiovascular parameters between men and women in the cohort. Especially for cholesterol & LDL-cholesterol, more aberrant measurements were observed for men than for women.

The detailed longitudinal visual exploration of the clinical data in Fig 4 was done to search for any (time-related) clinical events that could coincide/be related to the appearance of the physical disorders, as shown in Fig 3. Visually comparing the results of the clinical parameters (Fig 4) with the questionnaire parameters (Fig 3) did not immediately yield obvious one-on-one interactions. Further research will have to examine if predicting a significant correlation between the self-reported symptoms, clinical and omics parameters is possible.

### Genetic results

Whole-genome sequencing (WGS) was performed for all 30 participants to detect variants such as single-nucleotide polymorphisms (SNPs), and insertions and deletions (INDELs) [28] that could be associated with chronic diseases or illness indicators. On average, 3,807,610 SNPs and 855,955 INDELs were identified for each participant. Of these variants, 94.43% were registered in the database for single nucleotide polymorphism dbSNP (build 138) [29]. Out of all SNPs analyzed, there were 11,342 synonymous, 11,597 missense, 168 stop gain, 61 stop loss, and 38 start loss mutations.

Next, the participants’ genome results were compared with the ACMG SF v2.0 list, a list of 59 genes for which it is considered clinically evident that pathogenic variants of the genes result in diseases that might be prevented or treated. This resulted in two hits [19]. One person had a pathogenic variant of the BRCA2 gene (rs28897756) related to breast cancer. The other person had mutations in the MUTYH gene (rs34612342) related to colorectal cancer. Communication of those genetic IFs towards the participants was handled according to the strategy described earlier in the feedback to participants’ section. A qualitative evaluation of this communication strategy was investigated through the WGS perception questionnaire study.

### Genetic background

For quality control, the genetic structure of the IAM Frontier cohort was compared with other populations worldwide (S4 Fig). A principal component analysis (PCA) was used for this endeavor, with the identified variants in the whole-genome sequences and the 1000 Genomes Project as a reference panel. Only SNPs located on autosomes with a minor allele frequency above 5% and a linkage disequilibrium of less than 0.3 were considered in the analysis. The first two components captured the main variance with 48.55% and 23.88%, respectively. Using these two components, the 30 participants of the IAM Frontier study were mapped as a European population with overlaps withn admixed populations, as seen in S4A Fig.

PCA was repeated but only with European sub-populations in the 1000 Genomes Project using the same set of filtered SNPs as described above to see the differences between other European populations. S4B Fig presents the results, where most participants were mapped between the British and Scottish Population and Utah residents with European ancestry. These findings support the validity of the genomics data, as all IAM Frontier participants were reported to have European backgrounds, based on the information obtained from the self-reported ethnic background questionnaire.

### Multi-omics results

**Fig 5:**
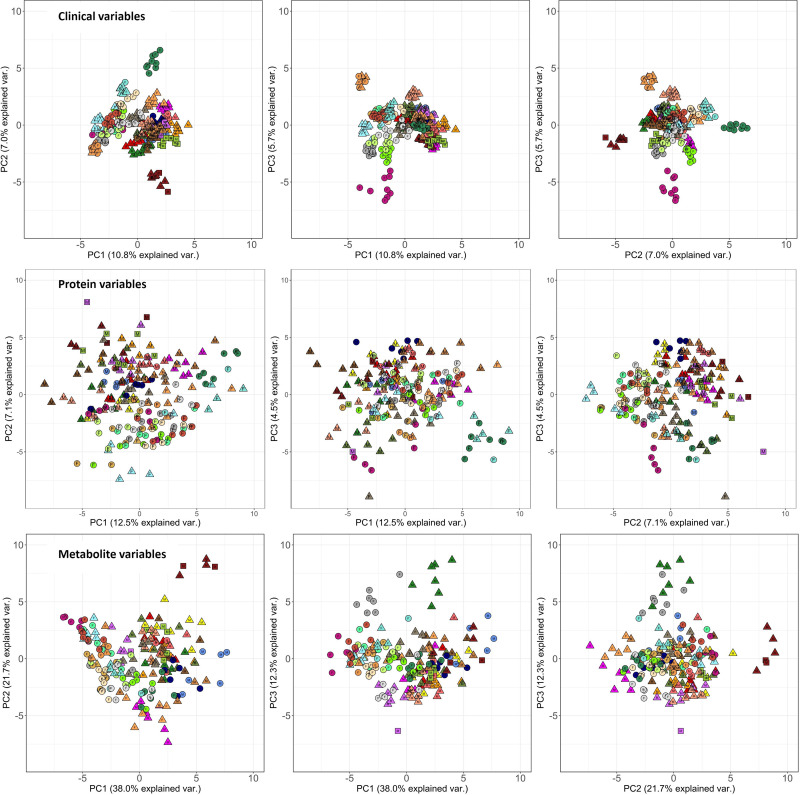
PCA plots of the various longitudinal omics datatypes. The three principal components (PC) that capture most of the variance in each data type are plotted, resulting in three possible combinations to plot these PC’s on a one-to-one basis. A) clinome data, B) proteomics, C) metabolomics, annotated with gender (M = Male, F = Female) and BMI information. Each point in the plot represents an individual at one specific time point, different measurements from a same individual are represented in one colour. The longitudinal character of the I AM Frontier data reveals that clinical parameters behave more stable over time and produce more dense inter-personal clusters compared to proteomics and metabolomics data. All 30 individuals are rep-resented by a distinct colour. O = BMI < 25, △ = 25 > BMI < 30, 2 = BMI > 30.

Fig 5 gives some preliminary information on the diverse data collection and the longitudinal robustness of the cohort study participants’ clinical biochemistry, proteomics, and metabolomics measurements, respectively. The various targeted parameters represent the biomolecular entities of the collected bio-samples. It is generally accepted that these entities reside in the same cell environments and affect the same clinical endpoint/phenotype [30]. However, Fig 5 shows that these somehow intertwined data layers have different longitudinal stability within and across individuals. For the clinical variables, measurements remain relatively constant over time compared to the protein and metabolite variables. Various data pre-processing steps and batch corrections were performed for the different data types (see data analysis techniques). Thus, we could rule out that this difference in longitudinal stability between different types of omics data was due to variations in the technical sampling process.

## Discussion

The I AM Frontier cohort is a proof-of-concept study in Flanders, Europe, that aims to support the development of a precision health-driven healthcare system, with a personalized approach to disease prevention. The study was designed to identify patterns, markers and processes that play a role in the spectrum between health and early onset of disease by combining information from a clinical setting with multi-omics, questionnaires and continuous personal monitoring data.

Our preliminary findings in this cohort, which consists of healthy people without disease diagnoses, showed that physical disorders are frequently present but not at a dysfunctional level (S1 Fig) and that cardiovascular risk is, for example, increased in men (S1 Table), presumably due to increased BMI and cholesterol levels. Preliminary results indicated that self-reported physical symptoms did not coincide with clinical events. Still, more than half of participants reported symptoms such as sleep problems and back pain, with 20% rating their sleep issues as serious. The metabolic symptoms cluster showed the highest burden, and certain symptoms—like headaches—were more common in women. Symptom reporting remained consistent over time, though some, like indigestion and muscle pain, varied in severity; seasonal trends were also evident, particularly in Cold/Flu symptoms. This finding, and the combination of multiple omics datasets, which contain more biological detail than the clinical measurements, will be further explored.

Genetic analyses revealed the presence of pathogenic variants in members of the cohort who could benefit from early disease detection. Evaluation on how participants experienced these results will be reported on in the future. Nonetheless, based on these results, it seems that other types of molecular entity parameters, like genomics, proteomics, metabolomics, etc., have significant added value compared to the currently established clinical data.

In the context of longitudinal data collection, a noteworthy observation is the inconvenience of a large sample donation at clinical institutes and the level of discomfort (e.g., from venipuncture) experienced by participants. Although in our cohort we did not experience drop-outs as a result of this, in a larger trial, this is expected to be a threshold for people to participate, thereby affecting the long-term viability of precision health cohorts. Therefore, efforts towards multi-omics micro sampling methods that retain sample quality will be vital in the future. Very recently, a robust microsampling approach was presented that allows individuals to collect small volume blood samples at home by themselves. As sample quality and parameter depth are not compromised, such an approach is a promising opportunity to facilitate sample collection and increase participant compliance in precision health cohort studies [10]. Other cohort studies can adopt this microsampling approach and use findings of the IAF cohort analyses to assess the minimum (longitudinal) collection frequency and specific omics variables that offer added value for detecting early signs of health deviation. Thereby, the IAF cohort can support and contribute in the long run to the evolution towards a personalized prevention approach in a clinical setting [31].

The intention is to link and integrate the IAF data with publicly available datasets to enrich personal health data of the IAF cohort in terms of numbers and provide context in terms of disease phenotypes, enabling a wider range of analyses. As this was anticipated, the sampling technologies used in the IAF cohort were selected to maximize overlap with large cross-sectional biobanks, such as the UK Biobank [32]. IAF participants agreed to the retention of data for a period of 20 years through an informed consent. Due to the limited number of participants, the IAF cohort data is currently only available for further research upon reasonable request. Requests will be processed on a study specific basis. The privacy sensitive nature of the data did not allow it to be deposited into public repositories [33].

Our future trajectory builds upon the foundation established by the IAF cohort. Ongoing analyses of this cohort are providing valuable insights into various key health parameters and disease risk factors. To scale up these efforts, an online web application containing health promoting tools was set up [34]. One such tool is the Domus Medica Health Guide, developed to provide individuals with a personalized risk analysis for chronic diseases, based on their lifestyle and anamnesis. The platform enables individuals to identify their health risks, obtain a personalized action plan to act upon these health risks, and share their health-related lifestyle data with their general practitioners and researchers. Currently, 1,700 individuals are already enrolled (Van den Eynde et al., in preparation). The Domus Medica Health Guide aims to set up a large-scale cohort that can be followed up over time. In the future, individuals could be contacted to participate in follow-up research where we introduce the markers selected in the I AM Frontier study.

By integrating findings from the IAF cohort into a large-scale cohort on personalized prevention, and incorporating new technologies for microsample data collection and participant engagement, we can develop more precise and individualized health recommendations. As we move forward, the expansion phase awaits fine-tuning, but this approach allows us to refine personalized prevention strategies and guide the design of future large-scale population health cohorts. This integrated approach will enhance our capacity for data expansion and enable us to make significant strides in improving public health outcomes.

## Conclusion

The I AM frontier cohort consists of deeply phenotyped patient samples with a profound sampling density (over 200 clinical parameters as well as omics technologies in blood, urine, stool, saliva as well as data collection through wearables, questionnaires, and interviews) and unique longitudinal granularity. Therefore, the development of future precision health applications and the prevention of chronic diseases through prospective research can benefit from the availability of this biobank.

One limitation of this study design was that data collection had to be stopped after 12 months, six months earlier than planned, due to the COVID-19 pandemic that arose in 2020, but this had no impact on the first year of data collection. We did not have any drop-outs in the first year. Only in the very last month of March 2020 ten drop-outs occurred. Through questionnaires, participants reported that our approach of giving feedback on certain parts of the results helped maintain their motivation to participate in the study.

### Strengths and limitations summary

• The I AM Frontier proof-of-concept (POC) cohort study is unique in collecting an extensive range of samples, with high longitudinal frequency, of healthy individuals for 12 months. The implemented sampling technologies (for clinical parameters, whole genome sequencing (WGS), methylation, quantitative proteomics, metabolomics, microbiome, retina scans, wearables, and standardized questionnaires on, e.g., food intake and medical status in combination with genome sequencing at the start of the study) were selected to maximize overlap with large cross-sectional studies and biobanks such as UK biobank to allow comparison of phenotypical profiles present across different studies.
• The highly granular (i.e., collected with high longitudinal frequency) data within this study allows us to construct dense participant profiles. Frequent longitudinal data collection of multi-omics data is emerging with new technical advancements for the in-depth analyses of molecules in small blood volumes. To allow the routine usage of such measurements in clinical practice, the temporal changes observed in this cohort can serve to evaluate the frequency and added value of such highly granular measurements.
• Perceptions of precision health, such as communication of clinical follow-up data, personal risks from genomics, behavioral aspects, and the ethical dilemmas that go together with all of this, are included in the scope of the cohort.

## Supporting information

S1 Fig Baseline physical symptoms of IAM Frontier participants obtained at the time of recruitment with the CHIPS methodology from 0 (in green: the least severe/painless) to 4 (in red: severe pain). The horizontal width of each colored bar indicates how many participants indicate to suffer from that specific physical symptom with the corresponding severity.
(TIF)

S2 Fig Physical symptoms throughout the study period (timepoints 0–12) were obtained via questionnaires and grouped according to the findings of Allen et. al (22). The color intensity indicates the severity of the complaint on a scale from zero to four. Month zero is March 2019, and month 12 is March 2020. All time points are shown for the female participants.
(TIF)

S3 Fig Longitudinal heatmap representing which clinical parameters (right), grouped in clinical health clusters (left), are out of normal range bounds, i.e., population reference intervals (PRI), for female participants. (TIF)

S4 Fig A) Genetic structure of I AM Frontier cohort as red points compared with worldwide populations, B) and with only European sub-populations. (TIF)

S1 Table Supplementary table Clinicals. This table contains a variable-specific overview of all clinical variables measured in our cohort. (PDF)

S2 Table Supplementary table Proteomics. This table contains a variable-specific overview of all proteins measured in our cohort. (PDF)

S3 Table Supplementary table Metabolomics. This table contains a variable-specific overview of all metabolites measured in our cohort. (PDF)

S1 File Supplementary document 1. (PDF)

S2 File Supplementary document 2. (TIF)

## Abbreviations

ACMG: American College of Medical Genetics and Genomics
ADA: American diabetes association
BMI: Body mass index
CHIPS: Cohen-Hoberman Inventory of Physical Symptoms
CVD: Cardiovascular disease
EDTA: Ethylenediaminetetraacetic acid
GP: General practitioner
IPOP: Integrated personal omics profiling
MS: Mass spectrometry
NMR: Nuclear magnetic resonance
PBMC: Peripheral blood mononuclear cells
PCA: Principle component analyses
POC: Proof of concept
SNP: Single nucleotide polymorphism
WGS: Whole genome sequencing
