# Supplementary figures and images for: Cohort profile: The Belgian I AM frontier prospective cohort study for comprehensive health outcome exploration
Source: PLoS One. 2025 Jun 12;20(6):e0326024. doi: 10.1371/journal.pone.0326024 (PMC12161581; doi:10.1371/journal.pone.0326024)

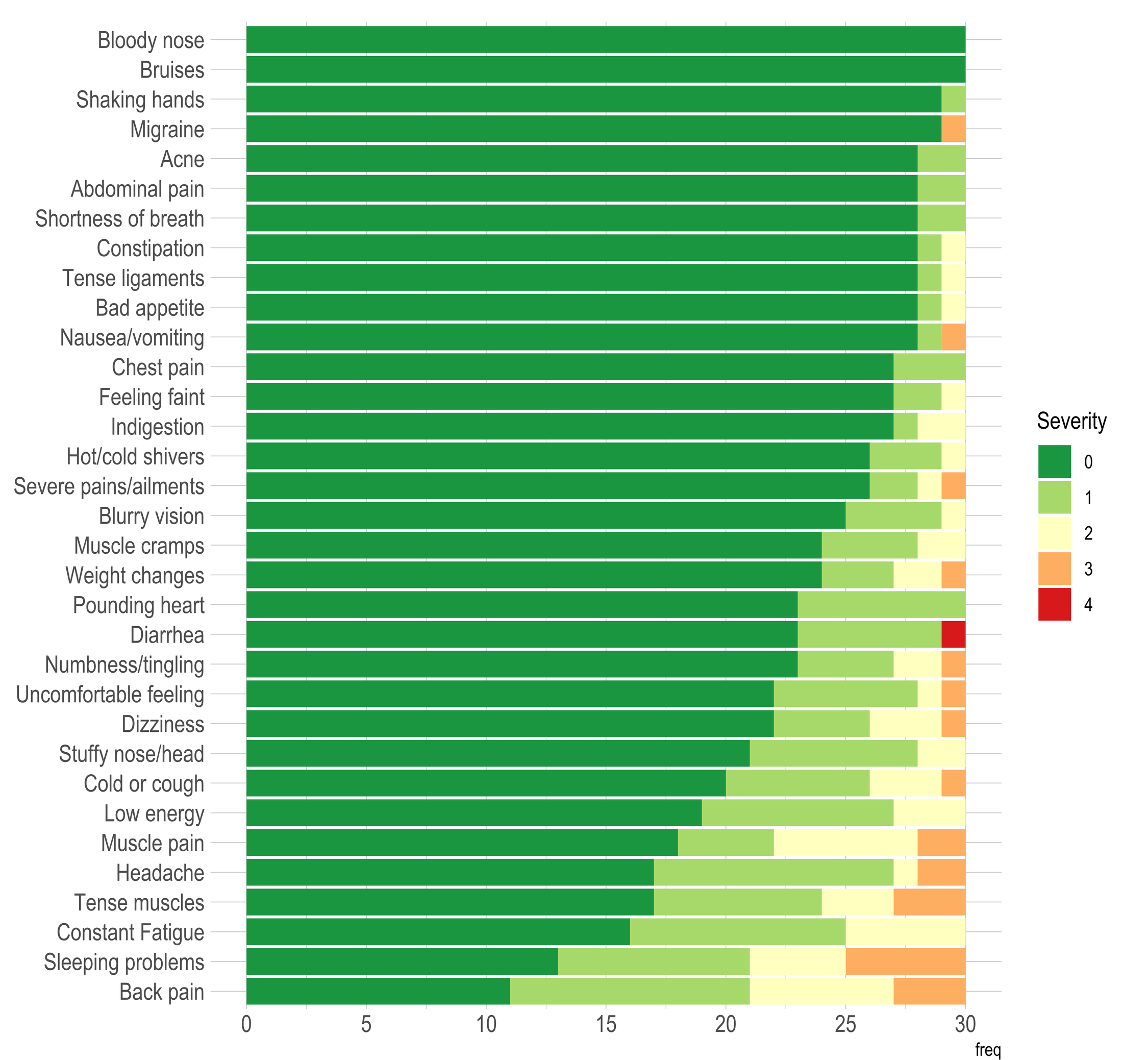

Supplement: S1 Fig — The horizontal width of each colored bar indicates how many participants indicate to suffer from that specific physical symptom with the corresponding severity. (TIF) [file pone.0326024.s001.tif]

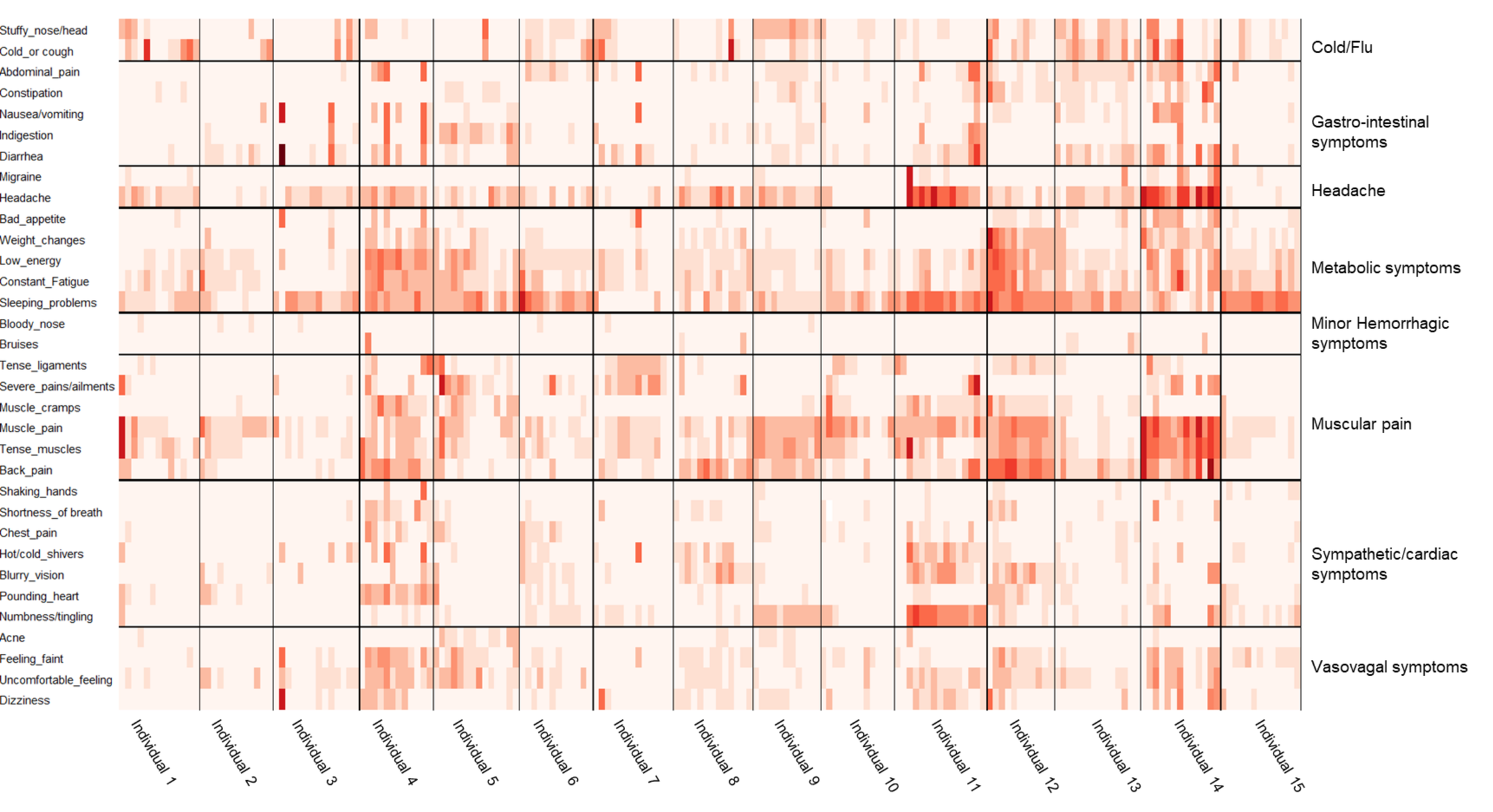

Supplement: S2 Fig — The color intensity indicates the severity of the complaint on a scale from zero to four. Month zero is March 2019, and month 12 is March 2020. All time points are shown for the female participants. (TIF) [file pone.0326024.s002.tif]

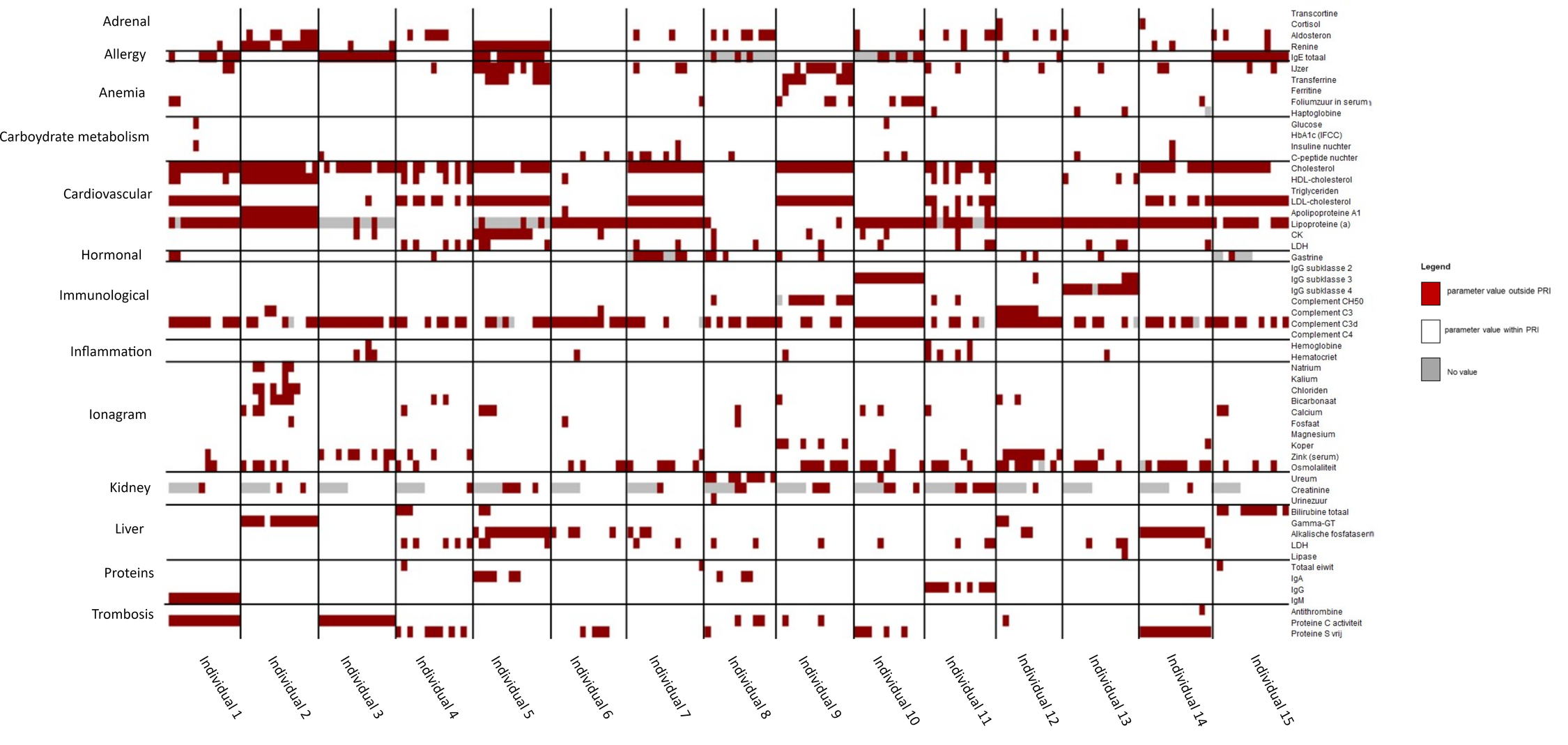

Supplement: S3 Fig — (TIF) [file pone.0326024.s003.tif]

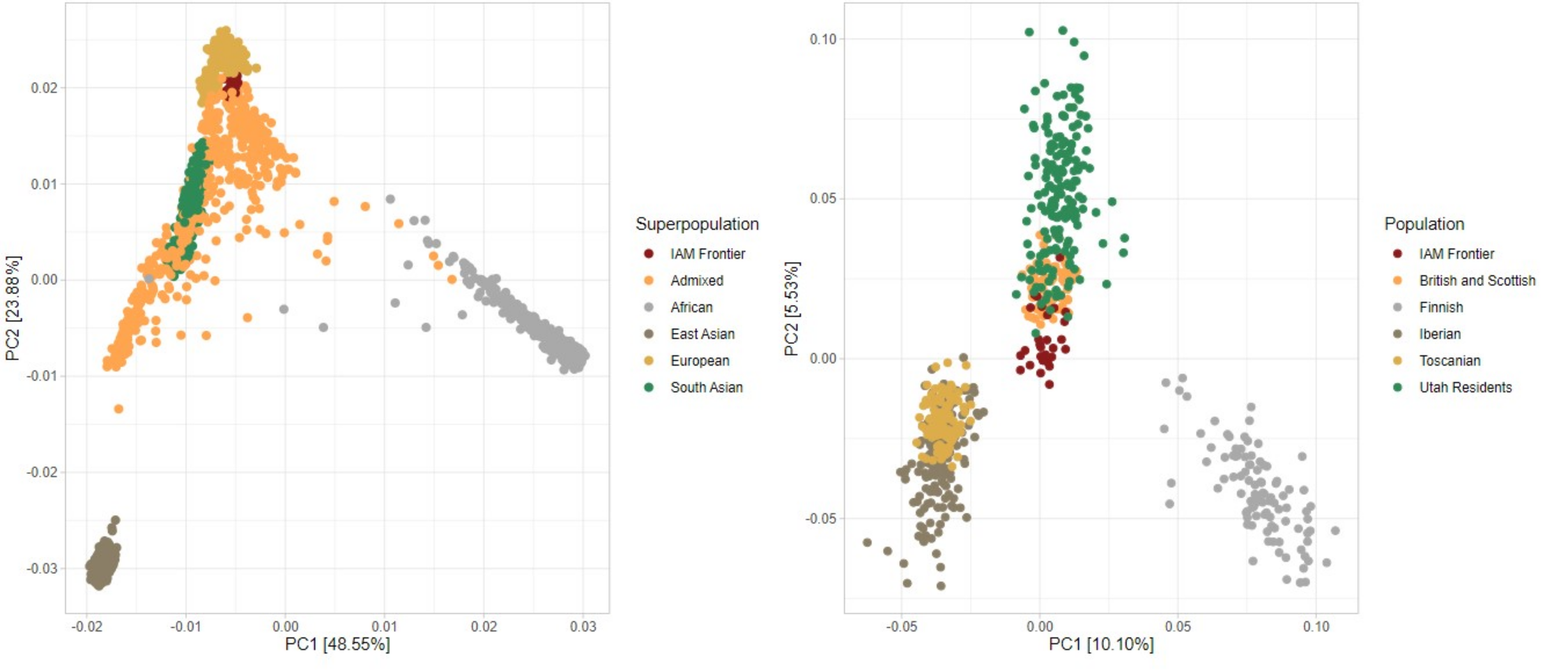

Supplement: S4 Fig — (TIF) [file pone.0326024.s004.tif]
