# Supplementary material for: Cohort profile: The Belgian I AM frontier prospective cohort study for comprehensive health outcome exploration
Source: PLoS One. 2025 Jun 12;20(6):e0326024. doi: 10.1371/journal.pone.0326024 (PMC12161581; doi:10.1371/journal.pone.0326024)
Supplement: S1 Table — (PDF) [file pone.0326024.s005.pdf]

Endpoint	Clinical parameters													Times tested
**Allergy**	gx3 Grasses	wx5 Herbs 1	wx6 Herbs 2	tx5 Trees 1	tx6 Trees 2	fx5 Food								1
**Allergy**	mx1 Molds	ex1 Animal dander	d1 House dust mite	h1 House dust (Greer)	h2 House dust (Hollister)	f1 Egg white	f2 Cow's milk	f4 Wheat	f13 Peanut	f14 Soybean	f79 Gluten	c1 Penicilloyl G	c2 Penicilloyl V	1
**Allergy**	IgE													8
**Anemia**	Iron	Transferrin	Ferritin	Folic acid	Haptoglobin									13
**Antibodies directed against**	Adrenal gland	Skin	Insulin	Pancreas	Ac. Chol. Rec	Smooth muscle	LKM	Mitochondria	Intrinsic factor	Kidney (GBM)				1
**Adrenal gland**	Transcortin	Cortisol	Aldosterone	Renin										13
**Bleeding**	Factor VIII	Factor IX	APTT	Fibrinogen	von Willebrand factor activity		Homocysteine							1
**Bone Metabolism**	Bone specific alkaline phosphatase		Parathyroid hormone											1
**Bone Metabolism**	Vitamin D													8
**Cardiovascular**	Cholesterol	HDL Cholesterol	Triglycerides	LDL Cholesterol	Apolipoprotein A1/B	LP(a)	CK	Troponin	LDH					13
**Celiac disease**	Transglutaminase IgA													1
**Hormones**	IGF-I	PSA secreening/free PSA (M)	Progesterone/DHEA sulfate/Testosterone/SHBG (M+F)		Estrone (F)									1
**Hormones**	Gastrin													13
**Immunohaematology & Immunology**	Blood type ABO-D E+S	Rhesus	Direct Antiglobulin	Irregular antibodies	Tryptase									1
**Immunology**	T, B, and NK cells	T cells, T4/T8												8
**Immunology**	IgG2	IgG3	IgG4	Complement CH50	Complement C3	Complement C3d	Complement C4							13
**Inflammation**	Sedimentation													8
**Inflammation**	Hemoglobin	Hematocrit	WBC	WBC formula	Platelets	Ultra sensitive CRP								13
**Ionogram**	Sodium	Potassium	Chlorides	Bicarbonate	Calcium	Phosphate	Magnesium	Copper	Zinc	Osmolality				13
**Carbohydrate metabolism**	Glucose (fasting)	HbA1c	Insuline (fasting)	C-peptide (fasting)										13
**Liver**	Bilirubin total	AST	ALT	Gamma-GT	Alkaline phosphatase	LDH	Lipase							13
**Kidney**	Urea	Creatinine	Uric acid											13
**Orthomolecular tests**	Glutathione peroxidase	Total antioxidant status												1
**Other**	Immunofixation	Calcitonin	ACTH	Angiotens. Conv. Enz. (ACE)	Cerulopasmin + copper	Cholinesterase	Transthyretin	Alph-1 antitrypsin	Beta-2 microglubulin	C1-inhibitor	BNP	TSI		1
**Proteins**	Protein total	Protein electrophoresis	IgA	IgG	IGM									13
**Rheumatism - autoantibodies**	Rheumatoid factor (RF)	Anti-CCP	Antinuclear antibodies (ANA)	HLA B27										1
**Thyroid**	TSH	Free T4	Anti-thyroglobulin	Thryoglobulin										8
**Thrombosis**	D-Dimers	APC-resistance	Anti-cardiolipin IgM	Anti-cardiolipin IgG	Anti-B2 GPI IgM	Anti-B2 GPI IgG								1
**Thrombosis**	Antithrombin	Protein C activity	Protein S free											13
**Vitamins**	Vitamin A	Vitamin C (**Month 4 and 10 only**)		Vitamin E	Vitamin B12	Vitamin B1	Vitamin B2	Vitamin B6	B-Carotene					8

Color Code to distribute single time tests over time	1st month	2nd month	4th month	6th month	10th month	12th month
