# Supplementary material for: Cohort profile: The Belgian I AM frontier prospective cohort study for comprehensive health outcome exploration
Source: PLoS One. 2025 Jun 12;20(6):e0326024. doi: 10.1371/journal.pone.0326024 (PMC12161581; doi:10.1371/journal.pone.0326024)
Supplement: S2 Table — (PDF) [file pone.0326024.s006.pdf]

Protein assay list

Olink® Target 96 Cardiometabolic

Product number: 95360

Angiogenin (ANG)	P03950	Endoglin (ENG)	P17813
Angiopoietin-related protein 3 (ANGPTL3)	Q9Y5C1	Fetuin-B (FETUB)	Q9UGM5
Apolipoprotein M (APOM)	O95445	Ficolin-2 (FCN2)	Q15485
Beta-Ala-His dipeptidase (CNDP1)	Q96KN2	Glutaminyl-peptide cyclotransferase (QPCT)	Q16769
Beta-galactoside alpha-2,6-sialyltransferase 1 (ST6GAL1)	P15907	Granulysin (GNLY)	P22749
Cadherin-1 (CDH1)	P12830	Growth arrest-specific protein 6 (GAS6)	Q14393
Carbonic anhydrase 1 (CA1)	P00915	Hepatocyte growth factor receptor (MET)	P08581
Carbonic anhydrase 3 (CA3)	P07451	Ig lambda-2 chain C regions (IGLC2)	P0CG05
Carbonic anhydrase 4 (CA4)	P22748	Insulin-like growth factor-binding protein 3 (IGFBP3)	P17936
Cartilage acidic protein 1 (CRTAC1)	Q9NQ79	Insulin-like growth factor-binding protein 6 (IGFBP6)	P24592
Cartilage oligomeric matrix protein (COMP)	P49747	Integrin alpha-M (ITGAM)	P11215
C-C motif chemokine 5 (CCL5)	P13501	Intercellular adhesion molecule 1 (ICAM1)	P05362
C-C motif chemokine 14 (CCL14)	Q16627	Intercellular adhesion molecule 3 (ICAM3)	P32942
C-C motif chemokine 18 (CCL18)	P55774	Interleukin-7 receptor subunit alpha (IL7R)	P16871
CD59 glycoprotein (CD59)	P13987	Latent-transforming growth factor beta-binding protein 2 (LTBP2)	Q14767
Coagulation factor VII (F7)	P08709	Leukocyte immunoglobulin-like receptor subfamily B member 1 (LILRB1)	Q8NHL6
Coagulation factor XI (F11)	P03951	Leukocyte immunoglobulin-like receptor subfamily B member 2 (LILRB2)	Q8N423
Collagen alpha-1(XVIII) chain (COL18A1)	P39060	Leukocyte immunoglobulin-like receptor subfamily B member 5 (LILRB5)	O75023
Complement C1q tumor necrosis factor-related protein 1 (C1QTNF1)	Q9BXJ1	Lithostathine-1-alpha (REG1A)	P05451
Complement C2 (C2)	P06681	Liver carboxylesterase 1 (CES1)	P23141
Complement factor H-related protein 5 (CFHR5)	Q9BXR6	Low affinity immunoglobulin gamma Fc region receptor II-a (FCGR2A)	P12318
Complement receptor type 2 (CR2)	P20023	Low affinity immunoglobulin gamma Fc region receptor III-B (FCGR3B)	O75015
Cystatin-C (CST3)	P01034	L-selectin (SELL)	P14151
Dipeptidyl peptidase 4 (DPP4)	P27487		
EGF-containing fibulin-like extracellular matrix protein 1 (EFEMP1)	Q12805		

Table continues on reverse ▶

Lymphatic vessel endothelial hyaluronic acid receptor 1 (LYVE1)	Q9Y5Y7	Plexin-B2 (PLXNB2)	O15031
Lysosomal Pro-X carboxypeptidase (PRCP)	P42785	Procollagen C-endopeptidase enhancer 1 (PCOLCE)	Q15113
Mannose-binding protein C (MBL2)	P11226	Prolyl endopeptidase FAP (FAP)	Q12884
Mast/stem cell growth factor receptor Kit (KIT)	P10721	Receptor-type tyrosine-protein phosphatase S (PTPRS)	Q13332
Membrane cofactor protein (CD46)	P15529	Regenerating islet-derived protein 3-alpha (REG3A)	Q06141
Membrane primary amine oxidase (AOC3)	Q16853	Serum amyloid A-4 protein (SAA4)	P35542
Metalloproteinase inhibitor 1 (TIMP1)	P01033	SPARC-like protein 1 (SPARCL1)	Q14515
Microfibrillar-associated protein 5 (MFAP5)	Q13361	Superoxide dismutase [Cu-Zn] (SOD1)	P00441
Multiple epidermal growth factor-like domains protein 9 (MEGF9)	Q9H1U4	T-cell immunoglobulin and mucin domain-containing protein 4 (TIMD4)	Q96H15
Neural cell adhesion molecule 1 (NCAM1)	P13591	Tenascin (TNC)	P24821
Neural cell adhesion molecule L1-like protein (CHL1)	O00533	Tenascin-X (TNXB)	P22105
Neurogenic locus notch homolog protein 1 (NOTCH1)	P46531	Thrombospondin-4 (THBS4)	P35443
Neuropilin-1 (NRP1)	O14786	Thyroxine-binding globulin (SERPINA7)	P05543
Neutrophil defensin 1 (DEFA1)	P59665	Transcobalamin-2 (TCN2)	P20062
Neutrophil gelatinase-associated lipocalin (LCN2)	P80188	Transforming growth factor beta receptor type 3 (TGFBR3)	Q03167
Nidogen-1 (NID1)	P14543	Transforming growth factor-beta-induced protein ig-h3 (TGFBI)	Q15582
Oncostatin-M-specific receptor subunit beta (OSMR)	Q99650	Trypsin-2 (PRSS2)	P07478
Peptidyl-glycine alpha-amidating monooxygenase (PAM)	P19021	Tyrosine-protein kinase receptor Tie-1 (TIE1)	P35590
Phospholipid transfer protein (PLTP)	P55058	Uromodulin (UMOD)	P07911
Plasma serine protease inhibitor (SERPINA5)	P05154	Vascular cell adhesion protein 1 (VCAM1)	P19320
Platelet glycoprotein Ib alpha chain (GP1BA)	P07359	Vasorin (VASN)	Q6EMK4
Platelet-activating factor acetylhydrolase (PLA2G7)	Q13093	Vitamin K-dependent protein C (PROC)	P04070

For more details visit www.olink.com/cardiometabolic

www.olink.com

For research use only. Not for use in diagnostic procedures.
This product includes a license for non-commercial use. Commercial users may require additional licenses. Please contact Olink Proteomics AB for details.
There are no warranties, expressed or implied, which extend beyond this description. Olink Proteomics AB is not liable for property damage, personal injury, or economic loss caused by this product.
Olink® is a registered trademark of Olink Proteomics AB.

© 2017–2022 Olink Proteomics AB. All third party trademarks are the property of their respective owners.
Olink Proteomics, Dag Hammarskjölds väg 52B , SE-752 37 Uppsala, Sweden
1065, v2.0, 2022-06-14

Olink®

Protein assay list

Olink® Target 96 Cell Regulation

Product number: 95370

Amphoterin-induced protein 2 (AMIGO2)	Q86SJ2	Discoidin, CUB and LCCL domain-containing protein 2 (DCBLD2)	Q96PD2
Amyloid beta A4 precursor protein-binding family B member 1-interacting protein (APBB1IP)	Q7Z5R6	DnaJ homolog subfamily B member 1 (DNAJB1)	P25685
		Dual specificity mitogen-activated protein kinase kinase 6 (MAP2K6)	P52564
Anterior gradient protein 3 (AGR3)	Q8TD06	Dynactin subunit 2 (DCTN2)	Q13561
Arylsulfatase B (ARSB)	P15848	E3 ubiquitin-protein ligase CBL (CBL)	P22681
ATP-dependent 6-phosphofructokinase, muscle type (PFKM)	P08237	Ectonucleoside triphosphate diphosphohydrolase 6 (ENTPD6)	O75354
Bcl-2-like protein 11 isoform BimL (BCL2L11)	O43521	Fibroblast growth factor 21 (FGF21)	Q9NSA1
Beta-1,3-galactosyl-O-glycosyl-glycoprotein beta-1,6-N-acetylglucosaminyltransferase (GCNT1)	Q02742	Friend leukemia integration 1 transcription factor (FLI1)	Q01543
Biglycan (BGN)	P21810	Galectin-7 (LGALS7)	P47929
Bile salt sulfotransferase (SULT2A1)	Q06520	Gamma-secretase-activating protein (GSAP)	A4D1B5
Breakpoint cluster region protein (BCR)	P11274	Gastrokine (GKN1)	Q9NS71
Brother of CDO (BOC)	Q9BWV1	GDNF family receptor alpha-2 (GFRA2)	O00451
Calcium/calmodulin-dependent protein kinase kinase 1 (CAMKK1)	Q8N5S9	Glucagon (GCG)	P01275
Calsyntenin-3 (CLSTN3)	Q9BQT9		
Cell growth-regulating nucleolar protein (LYAR)	Q9NX58	Growth hormone variant (GH2)	P01242
Cellular tumor antigen p53 (TP53)	P04637	Heparan sulfate glucosamine 3-O-sulfotransferase 3B1 (HS3ST3B1)	Q9Y662
Cerebral dopamine neurotrophic factor (CDNF)	Q49AH0	Heparan-sulfate 6-O-sulfotransferase 1 (HS6ST1)	O60243
Collagen alpha-1(IV) chain (COL4A1)	P02462	Immunoglobulin superfamily member 3 (IGSF3)	O75054
Cone-rod homeobox protein (CRX)	O43186	Interleukin-17 receptor B (IL17RB)	Q9NRM6
Cryptic protein (CFC1)	P0CG37	Kallikrein-12 (KLK12)	Q9UKR0
C-type mannose receptor 2 (MRC2)	Q9UBG0	Kazal-type serine protease inhibitor domain-containing protein 1 (KAZALD1)	Q96I82
Cysteine protease ATG4A (ATG4A)	Q8WYN0	Leucine-rich repeat neuronal protein 1 (LRRN1)	Q6UXK5
Cysteine-rich secretory protein 2 (CRISP2)	P16562	Ly6/PLAUR domain-containing protein 1 (LYPD1)	Q8N2G4
Dickkopf-like protein 1 (DKKL1)	Q9UK85	Lymphoid-restricted membrane protein (LRMP)	Q12912

Table continues on reverse ▶

Methionine aminopeptidase 1D, mitochondrial (METAP1D)	Q6UB28	Semaphorin-4C (SEMA4C)	Q9C0C4
Myelin-oligodendrocyte glycoprotein (MOG)	Q16653	Serine/threonine-protein kinase PAK 4 (PAK4)	O96013
N(G),N(G)-dimethylarginine dimethylaminohydrolase 1 (DDAH1)	O94760	Sialic acid-binding Ig-like lectin 10 (SIGLEC10)	Q96LC7
NF-kappa-B inhibitor epsilon (NFKBIE)	O00221	Sialic acid-binding Ig-like lectin 6 (SIGLEC6)	O43699
Nicalin (NCLN)	Q969V3	SLAM family member 8 (SLAMF8)	Q9P0V8
Ninjurin-1 (NINJ1)	Q92982	SLIT and NTRK-like protein 2 (SLITRK2)	Q9H156
Nuclear factor of activated T-cells, cytoplasmic 1 (NFATC1)	O95644	SLIT and NTRK-like protein 6 (SLITRK6)	Q9H5Y7
Oligodendrocyte-myelin glycoprotein (OMG)	P23515	Src kinase-associated phosphoprotein 1 (SKAP1)	Q86WV1
Opticin (OPTC)	Q9UBM4	Syntaxin-6 (STX6)	O43752
Peroxiredoxin-6 (PRDX6)	P30041	Syntaxin-16 (STX16)	O14662
Podocalyxin-like protein 2 (PODXL2)	Q9NZ53	T-cell leukemia/lymphoma protein 1B (TCL1B)	O95988
Polypeptide N-acetylgalactosaminyltransferase 2 (GALNT2)	Q10471	Transcription factor AP-1 (JUN)	P05412
Probable carboxypeptidase X1 (CPXM1)	Q96SM3	Transforming acidic coiled-coil-containing protein 3 (TACC3)	Q9Y6A5
Prokineticin-1 (PROK1)	P58294	Tudor and KH domain-containing protein (TDRKH)	Q9Y2W6
Prolactin regulatory element-binding protein (PREB)	Q9HCU5	Tumor necrosis factor receptor superfamily member 10A (TNFRSF10A)	O00220
Protein FAM19A5 (FAM19A5)	Q7Z5A7	Tumor-associated calcium signal transducer 2 (TACSTD2)	P09758
Protein Wnt-9a (WNT9A)	O14904	Vascular endothelial growth factor D (VEGFD)	O43915
Protocadherin-17 (PCDH17)	O14917	Vesicle-associated membrane protein 5 (VAMP5)	O95183
Ras GTPase-activating-like protein IQGAP2 (IQGAP2)	Q13576	VPS10 domain-containing receptor SorCS2 (SORCS2)	Q96PQ0
Regulator of G-protein signaling 8 (RGS8)	P57771	Wiskott-Aldrich syndrome protein family member 1 (WASF1)	Q92558
Rho GTPase-activating protein 1 (ARHGAP1)	Q07960	Wiskott-Aldrich syndrome protein family member 3 (WASF3)	Q9UPY6
Rho guanine nucleotide exchange factor 12 (ARHGEF12)	Q9NZN5	Zinc finger and BTB domain-containing protein 16 (ZBTB16)	Q05516
Seizure 6-like protein 2 (SEZ6L2)	Q6UXD5	Zinc finger and BTB domain-containing protein 17 (ZBTB17)	Q13105

For more details visit www.olink.com/cell-regulation

www.olink.com

For research use only. Not for use in diagnostic procedures.
This product includes a license for non-commercial use. Commercial users may require additional licenses. Please contact Olink Proteomics AB for details.
There are no warranties, expressed or implied, which extend beyond this description. Olink Proteomics AB is not liable for property damage, personal injury, or economic loss caused by this product.
Olink® is a registered trademark of Olink Proteomics AB.

© 2017–2022 Olink Proteomics AB. All third party trademarks are the property of their respective owners.
Olink Proteomics, Dag Hammarskjölds väg 52B , SE-752 37 Uppsala, Sweden
1066, v2.0, 2022-06-14

Protein assay list

Olink® Target 96 Cardiovascular II

Product number: 95500

2,4-dienoyl-CoA reductase, mitochondrial (DECR1)	Q16698
A disintegrin and metalloproteinase with thrombospondin motifs 13 (ADAM-TS13)	Q76LX8
ADM (ADM)	P35318
Agouti-related protein (AGRP)	O00253
Alpha-L-iduronidase (IDUA)	P35475
Angiopoietin-1 (ANG-1)	Q15389
Angiopoietin-1 receptor (TIE2)	Q02763
Angiotensin-converting enzyme 2 (ACE2)	Q9BYF1
Bone morphogenetic protein 6 (BMP-6)	P22004
Brother of CDO (Protein BOC)	Q9BWV1
Carbonic anhydrase 5A, mitochondrial (CA5A)	P35218
Carcinoembryonic antigenrelated cell adhesion molecule 8 (CEACAM8)	P31997
Cathepsin L1 (CTSL1)	P07711
C-C motif chemokine 3 (CCL3)	P10147
C-C motif chemokine 17 (CCL17)	Q92583
CD40 ligand (CD40-L)	P29965
Chymotrypsin C (CTRC)	Q99895
C-X-C motif chemokine 1 (CXCL1)	P09341
Decorin (DCN)	P07585
Dickkopf-related protein 1 (Dkk-1)	O94907
Fatty acid-binding protein, intestinal (FABP2)	P12104
Fibroblast growth factor 21 (FGF-21)	Q9NSA1
Fibroblast growth factor 23 (FGF-23)	Q9GZV9
Follistatin (FS)	P19883
Galectin-9 (Gal-9)	O00182
Gastric intrinsic factor (GIF)	P27352
Gastrotropin (GT)	P51161
Growth hormone (GH)	P01241
Growth/differentiation factor 2 (GDF-2)	Q9UK05
Heat shock 27 kDa protein (HSP 27)	P04792
Heme oxygenase 1 (HO-1)	P09601
Hydroxyacid oxidase 1 (HAOX1)	Q9UJM8
Interleukin-1 receptor antagonist protein (IL-1ra)	P18510
Interleukin-1 receptor-like 2 (IL1RL2)	Q9HB29
Interleukin-4 receptor subunit alpha (IL-4RA)	P24394
Interleukin-6 (IL6)	P05231
Interleukin-17D (IL-17D)	Q8TAD2
Interleukin-18 (IL-18)	Q14116
Interleukin-27 (IL-27)	Q8NEV9, Q14213
Kidney Injury Molecule (KIM1)	Q96D42
Lactoylglutathione lyase (GLO1)	Q04760
Lectin-like oxidized LDL receptor 1 (LOX-1)	P78380
Leptin (LEP)	P41159
Lipoprotein lipase (LPL)	P06858
Low affinity immunoglobulin gamma Fc region receptor II-b (IgG Fc receptor II-b)	P31994
Lymphotactin (XCL1)	P47992
Macrophage receptor MARCO (MARCO)	Q9UEW3
Matrix metalloproteinase-7 (MMP-7)	P09237
Matrix metalloproteinase-12 (MMP-12)	P39900
Melusin (ITGB1BP2)	Q9UKP3

Table continues on reverse ▶

Natriuretic peptides B (BNP)	P16860	Serine protease 27 (PRSS27)	Q9BQR3
NF-kappa-B essential modulator (NEMO)	Q9Y6K9	Serine/threonine-protein kinase 4 (STK4)	Q13043
Osteoclast-associated immunoglobulin-like receptor (hOSCAR)	Q8IYS5	Serpin A12 (SERPINA12)	Q8IW75
Pappalysin-1 (PAPPA)	Q13219	SLAM family member 5 (CD84)	Q9UIB8
Pentraxin-related protein PTX3 (PTX3)	P26022	SLAM family member 7 (SLAMF7)	Q9NQ25
Placenta growth factor (PGF)	P49763	Sortilin (SORT1)	Q99523
Platelet-derived growth factor subunit B (PDGF subunit B)	P01127	Spondin-2 (SPON2)	Q9BUD6
Poly [ADP-ribose] polymerase 1 (PARP-1)	P09874	Stem cell factor (SCF)	P21583
Polymeric immunoglobulin receptor (PIgR)	P01833	Superoxide dismutase [Mn], mitochondrial (SOD2)	P04179
Programmed cell death 1 ligand 2 (PD-L2)	Q9BQ51	T-cell surface glycoprotein CD4 (CD4)	P01730
Proheparin-binding EGF-like growth factor (HB-EGF)	Q99075	Thrombomodulin TM	P07204
Pro-interleukin-16 (IL16)	Q14005	Thrombopoietin (THPO)	P40225
Prolargin (PRELP)	P51888	Thrombospondin-2 (THBS2)	P35442
Prostasin (PRSS8)	Q16651	Tissue factor (TF)	P13726
Protein AMBP (AMBP)	P02760	TNF-related apoptosis-inducing ligand receptor 2 (TRAIL-R2)	O14763
Proteinase-activated receptor 1 (PAR-1)	P25116	Tumor necrosis factor receptor superfamily member 10A (TNFRSF10A)	O00220
Protein-glutamine gamma-glutamyltransferase 2 (TGM2)	P21980	Tumor necrosis factor receptor superfamily member 11A (TNFRSF11A)	Q9Y6Q6
Proto-oncogene tyrosine-protein kinase Src (SRC)	P12931	Tumor necrosis factor receptor superfamily member 13B (TNFRSF13B)	O14836
P-selectin glycoprotein ligand 1 (PSGL-1)	Q14242	Tyrosine-protein kinase Mer (MERTK)	Q12866
Receptor for advanced glycosylation end products (RAGE)	Q15109	Vascular endothelial growth factor D (VEGFD)	O43915
Renin (REN)	P00797	V-set and immunoglobulin domain-containing protein 2 (VSIG2)	Q96IQ7

For more details visit www.olink.com/cvd2

www.olink.com

For research use only. Not for use in diagnostic procedures.
This product includes a license for non-commercial use. Commercial users may require additional licenses. Please contact Olink Proteomics AB for details.
There are no warranties, expressed or implied, which extend beyond this description. Olink Proteomics AB is not liable for property damage, personal injury, or economic loss caused by this product.
Olink® is a registered trademark of Olink Proteomics AB.

© 2017–2022 Olink Proteomics AB. All third party trademarks are the property of their respective owners.
Olink Proteomics, Dag Hammarskjölds väg 52B , SE-752 37 Uppsala, Sweden
1024, v2.0, 2022-06-14

Olink®

Protein assay list

Olink® Target 96 Cardiovascular III

Product number: 95611

Aminopeptidase N (AP-N)	P15144	Galectin-3 (Gal-3)	P17931
Azurocidin (AZU1)	P20160	Galectin-4 (Gal-4)	P56470
Bleomycin hydrolase (BLM hydrolase)	Q13867	Granulins (GRN)	P28799
Cadherin-5 (CDH5)	P33151	Growth/differentiation factor 15 (GDF-15)	Q99988
Carboxypeptidase A1 (CPA1)	P15085	Insulin-like growth factor-binding protein 1 (IGFBP-1)	P08833
Carboxypeptidase B (CPB1)	P15086	Insulin-like growth factor-binding protein 2 (IGFBP-2)	P18065
Caspase-3 (CASP-3)	P42574	Insulin-like growth factor-binding protein 7 (IGFBP-7)	Q16270
Cathepsin D (CTSD)	P07339	Integrin beta-2 (ITGB2)	P05107
Cathepsin Z (CTSZ)	Q9UBR2	Intercellular adhesion molecule 2 (ICAM-2)	P13598
C-C motif chemokine 15 (CCL15)	Q16663	Interleukin-1 receptor type 1 (IL-1RT1)	P14778
C-C motif chemokine 16 (CCL16)	O15467	Interleukin-1 receptor type 2 (IL-1RT2)	P27930
C-C motif chemokine 24 (CCL24)	O00175	Interleukin-2 receptor subunit alpha (IL2-RA)	P01589
CD166 antigen (ALCAM)	Q13740	Interleukin-6 receptor subunit alpha (IL-6RA)	P08887
Chitinase-3-like protein 1 (CHI3L1)	P36222	Interleukin-17 receptor A (IL-17RA)	Q96F46
Chitotriosidase-1 (CHIT1)	Q13231	Interleukin-18-binding protein (IL-18BP)	O95998
Collagen alpha-1(I) chain (COL1A1)	P02452	Junctional adhesion molecule A (JAM-A)	Q9Y624
Complement component C1q receptor (CD93)	Q9NPY3	Kallikrein-6 (KLK6)	Q92876
Contactin-1 (CNTN1)	Q12860	Low-density lipoprotein receptor (LDL receptor)	P01130
C-X-C motif chemokine 16 (CXCL16)	Q9H2A7	Lymphotoxin-beta receptor (LTBR)	P36941
Cystatin-B (CSTB)	P04080	Matrix extracellular phosphoglycoprotein (MEPE)	Q9NQ76
Elafin (PI3)	P19957	Matrix metalloproteinase-2 (MMP-2)	P08253
Ephrin type-B receptor 4 (EPHB4)	P54760	Matrix metalloproteinase-3 (MMP-3)	P08254
Epidermal growth factor receptor (EGFR)	P00533	Matrix metalloproteinase-9 (MMP-9)	P14780
Epithelial cell adhesion molecule (Ep-CAM)	P16422	Metalloproteinase inhibitor 4 (TIMP4)	Q99727
E-selectin (SELE)	P16581	Monocyte chemotactic protein 1 (MCP-1)	P13500
Fatty acid-binding protein, adipocyte (FABP4)	P15090	Myeloblastin (PRTN3)	P24158

Table continues on reverse ▶

Myeloperoxidase (MPO)	P05164	Secretoglobin family 3A member 2 (SCGB3A2)	Q96PL1
Myoglobin (MB)	P02144	Spondin-1 (SPON1)	Q9HCB6
Neurogenic locus notch homolog protein 3 (Notch 3)	Q9UM47	ST2 protein (ST2)	Q01638
N-terminal prohormone brain natriuretic peptide (NT-proBNP)	NA	Tartrate-resistant acid phosphatase type 5 (TR-AP)	P13686
Osteopontin (OPN)	P10451	Tissue factor pathway inhibitor (TFPI)	P10646
Osteoprotegerin (OPG)	O00300	Tissue-type plasminogen activator (t-PA)	P00750
Paraoxonase (PON3)	Q15166	Transferrin receptor protein 1 (TR)	P02786
Peptidoglycan recognition protein 1 (PGLYRP1)	O75594	Trefoil factor 3 (TFF3)	Q07654
Perlecan (PLC)	P98160	Trem-like transcript 2 protein (TLT-2)	Q5T2D2
Plasminogen activator inhibitor 1 (PAI)	P05121	Tumor necrosis factor ligand superfamily member 13B (TNFSF13B)	Q9Y275
Platelet endothelial cell adhesion molecule (PECAM-1)	P16284	Tumor necrosis factor receptor 1 (TNF-R1)	P19438
Platelet-derived growth factor subunit A (PDGF subunit A)	P04085	Tumor necrosis factor receptor 2 (TNF-R2)	P20333
Platelet glycoprotein VI (GP6)	Q9HCN6	Tumor necrosis factor receptor superfamily member 6 (FAS)	P25445
Proprotein convertase subtilisin/kexin type 9 (PCSK9)	Q8NBP7	Tumor necrosis factor receptor superfamily member 10C (TNFRSF10C)	O14798
Protein delta homolog 1 (DLK-1)	P80370	Tumor necrosis factor receptor superfamily member 14 (TNFRSF14)	Q92956
P-selectin (SELP)	P16109	Tyrosine-protein kinase receptor UFO (AXL)	P30530
Pulmonary surfactant-associated protein D (PSP-D)	P35247	Tyrosine-protein phosphatase non-receptor type substrate 1 (SHPS-1)	P78324
Resistin (RETN)	Q9HD89	Urokinase plasminogen activator surface receptor (U-PAR)	Q03405
Retinoic acid receptor responder protein 2 (RARRES2)	Q99969	Urokinase-type plasminogen activator (uPA)	P00749
Scavenger receptor cysteine-rich type 1 protein M130 (CD163)	Q86VB7	von Willebrand factor (vWF)	P04275

For more details visit www.olink.com/cvd3

www.olink.com

For research use only. Not for use in diagnostic procedures.
This product includes a license for non-commercial use. Commercial users may require additional licenses. Please contact Olink Proteomics AB for details.
There are no warranties, expressed or implied, which extend beyond this description. Olink Proteomics AB is not liable for property damage, personal injury, or economic loss caused by this product.
Olink® is a registered trademark of Olink Proteomics AB.

© 2017–2022 Olink Proteomics AB. All third party trademarks are the property of their respective owners.

Olink Proteomics, Dag Hammarskjölds väg 52B , SE-752 37 Uppsala, Sweden
1023, v2.0, 2022-06-03

Olink®

Protein assay list

Olink® Target 96 Development

Product number: 95352

ADP-ribosyl cyclase/cyclic ADP-ribose hydrolase 2 (BST1)	Q10588	Contactin-4 (CNTN4)	Q8IWV2
ADP-sugar pyrophosphatase (NUDT5)	Q9UKK9	Corticotropin-releasing factor-binding protein (CRHBP)	P24387
Aggrecan core protein (ACAN)	P16112	C-type lectin domain family 11 member A (CLEC11A)	Q9Y240
Amyloid beta A4 protein (APP)	P05067	C-type lectin domain family 14 member A (CLEC14A)	Q86T13
Angiopoietin-related protein 4 (ANGPTL4)	Q9BY76	Cystatin-M (CST6)	Q15828
Arylsulfatase A (ARSA)	P15289	Cysteine-rich motor neuron 1 protein (CRIM1)	Q9NZV1
Basal cell adhesion molecule (BCAM)	P50895	Cysteine-rich with EGF-like domain protein 2 (CRELD2)	Q6UXH1
Beta-1,4-galactosyltransferase 1 (B4GALT1)	P15291	Cytosolic phospholipase A2 (PLA2G4A)	P47712
Beta-1,4-glucuronyltransferase 1 (B4GAT1)	O43505	Desmocollin-2 (DSC2)	Q02487
Beta-glucuronidase (GUSB)	P08236	Dickkopf-related protein 3 (DKK3)	Q9UBP4
Beta-microseminoprotein (MSMB)	P08118	Dystroglycan (DAG1)	Q14118
Carbonic anhydrase 2 (CA2)	P00918	Early activation antigen CD69 (CD69)	Q07108
Carbonic anhydrase 6 (CA6)	P23280	Ectonucleotide pyrophosphatase/phosphodiesterase family member 2 (ENPP2)	Q13822
Cathepsin F (CTSF)	Q9UBX1	Endothelial cell-selective adhesion molecule (ESAM)	Q96AP7
Cation-independent mannose-6-phosphate receptor (IGF2R)	P11717	Fc receptor-like protein 5 (FCRL5)	Q96RD9
C-C motif chemokine 21 (CCL21)	O00585	Flavin reductase NADPH (BLVRB)	P30043
CCN family member 5 (CCN5)	O76076	Follistatin-related protein 3 (FSTL3)	O95633
CD97 antigen (CD97)	P48960	Galactoside 3(4)-L-fucosyltransferase, Alpha-(1,3)-fucosyltransferase 3/5 (FUT3/5)	P21217, Q11128
CD99 antigen-like protein 2 (CD99L2)	Q8TCZ2	Glycoprotein hormones alpha chain (CGA)	P01215
CD109 antigen (CD109)	Q6YHK3	Glycoprotein Xg (XG)	P55808
CD177 antigen (CD177)	Q8N6Q3	Hepatitis A virus cellular receptor 2 (HAVCR2)	Q8TDQ0
CD209 antigen (CD209)	Q9NNX6	HLA class II histocompatibility antigen gamma chain (CD74)	P04233
Cell adhesion molecule-related/down-regulated by oncogenes (CDON)	Q4KMG0	Inactive serine protease PAMR1 (PAMR1)	Q6UXH9
CMRF35-like molecule 9 (CD300LG)	Q6UXG3	Inhibin beta C chain (INHBC)	P55103
Cochlin (COCH)	O43405	Integrin alpha-5 (ITGA5)	P08648
Collectin-12 (COLEC12)	Q5KU26	Integrin beta-1 (ITGB1)	P05556

Table continues on reverse ▶

Interleukin-13 receptor subunit alpha-1 (IL13RA1)	P78552
Kunitz-type protease inhibitor 1 (SPINT1)	O43278
Kunitz-type protease inhibitor 2 (SPINT2)	O43291
Lactadherin (MFGE8)	Q08431
Laminin subunit alpha-4 (LAMA4)	Q16363
LDLR chaperone MESD (MESDC2)	Q14696
Legumain (LGMN)	Q99538
Leukocyte-associated immunoglobulin-like receptor 1 (LAIR1)	Q6GTX8
Low affinity immunoglobulin epsilon Fc receptor (FCER2)	P06734
Lymphocyte function-associated antigen 3 (CD58)	P19256
Macrophage migration inhibitory factor (MIF)	P14174
Matrilin-2 (MATN2)	O00339
Myocilin (MYOC)	Q99972
Nidogen-2 (NID2)	Q14112
Osteomodulin (OMD)	Q99983
Paired immunoglobulin-like type 2 receptor alpha (PILRA)	Q9UKJ1
Peptidyl-prolyl cis-trans isomerase B (PPIB)	P23284
Phosphatidylethanolamine-binding protein 1 (PEBP1)	P30086
Platelet endothelial aggregation receptor 1 (PEAR1)	Q5VY43
Platelet-derived growth factor receptor beta (PDGFRB)	P09619
Protein deglycase DJ-1 (PARK7)	Q99497
Protein disulfide-isomerase (P4HB)	P07237
Protein NOV homolog (NOV)	P48745
Receptor-type tyrosine-protein phosphatase F (PTPRF)	P10586
Roundabout homolog 1 (ROBO1)	Q9Y6N7
Scavenger receptor class F member 1 (SCARF1)	Q14162
Semaphorin-7A (SEMA7A)	O75326
Serine protease HTRA2, mitochondrial (HTRA2)	O43464
Serine protease inhibitor Kazal-type 1 (SPINK1)	P00995
Serine protease inhibitor Kazal-type 5 (SPINK5)	Q9NQ38
Signal-regulatory protein beta-1 (SIRPB1)	O00241
Stress-induced-phosphoprotein 1 (STIP1)	P31948
Synaptosomal-associated protein 29 (SNAP29)	O95721
Thymosin beta-10 (TMSB10)	P63313
Tissue alpha-L-fucosidase (FUCA1)	P04066
Tripeptidyl-peptidase 1 (TPP1)	O14773
Tumor necrosis factor receptor superfamily member 19L (RELT)	Q969Z4
Tyrosine-protein phosphatase non-receptor type 6 (PTPN6)	P29350
WAP, Kazal, immunoglobulin, Kunitz and NTR domain-containing protein 2 (WFIKKN2)	Q8TEU8
V-set and immunoglobulin domain-containing protein 4 (VSIG4)	Q9Y279

For more details visit www.olink.com/development

www.olink.com

For research use only. Not for use in diagnostic procedures.
This product includes a license for non-commercial use. Commercial users may require additional licenses. Please contact Olink Proteomics AB for details.
There are no warranties, expressed or implied, which extend beyond this description. Olink Proteomics AB is not liable for property damage, personal injury, or economic loss caused by this product.
Olink® is a registered trademark of Olink Proteomics AB.

© 2017–2022 Olink Proteomics AB. All third party trademarks are the property of their respective owners.
Olink Proteomics, Dag Hammarskjölds väg 52B , SE-752 37 Uppsala, Sweden
1064, v2.0, 2022-06-14

Olink®

Protein assay list

Olink® Target 96 Immune Response

Product number: 95320

Allergin-1 (MILR1)	Q7Z6M3	Eotaxin (CCL11)	P51671
Amphiregulin (AR) (AREG)	P15514	Eukaryotic translation initiation factor 4 gamma 1 (EIF4G1)	Q04637
Aryl hydrocarbon receptor nuclear translocator (ARNT)	P27540	Eukaryotic translation initiation factor 5A-1 (EIF5A)	P63241
Baculoviral IAP repeat-containing protein 2 (BIRC2)	Q13490	Fc receptor-like protein 3 (FCRL3)	Q96P31
Beta-galactosidase (GLB1)	P16278	Fc receptor-like protein 6 (FCRL6)	Q6DN72
Butyrophilin subfamily 3 member A2 (BTN3A2)	P78410	Fibroblast growth factor 2 (FGF2)	P09038
CD83 antigen (CD83)	Q01151	FXYD domain-containing ion transport regulator 5 (FXYD5)	Q96DB9
Contactin-associated protein-like 2 (CNTNAP2)	Q9UHC6	Hematopoietic lineage cell-specific protein (HCLS1)	P14317
Corneodesmosin (CDSN)	Q15517	Histamine N-methyltransferase (HNMT)	P50135
Corticosteroid 11-beta-dehydrogenase isozyme 1 (HSD11B1)	P28845	Importin subunit alpha-5 (KPNA1)	P52294
Coxsackievirus and adenovirus receptor (CXADR)	P78310	Inactive dipeptidyl peptidase 10 (DPP10)	Q8N608
C-type lectin domain family 4 member A (CLEC4A)	Q9UMR7	Integral membrane protein 2A (ITM2A)	O43736
C-type lectin domain family 4 member C (CLEC4C)	Q8WTT0	Integrin alpha-6 (ITGA6)	P23229
C-type lectin domain family 4 member D (CLEC4D)	Q8WXI8	Integrin alpha-11 (ITGA11)	Q9UKX5
C-type lectin domain family 4 member G (CLEC4G)	Q6UXB4	Integrin beta-6 (ITGB6)	P18564
C-type lectin domain family 6 member A (CLEC6A)	Q6EIG7	Interferon lambda receptor 1 (IFNLR1)	Q8IU57
C-type lectin domain family 7 member A (CLEC7A)	Q9BXN2	Interferon regulatory factor 9 (IRF9)	Q00978
Cytoskeleton-associated protein 4 (CKAP4)	Q07065	Interleukin-1 receptor-associated kinase 1 (IRAK1)	P51617
Diacylglycerol kinase zeta (DGKZ)	Q13574	Interleukin-1 receptor-associated kinase 4 (IRAK4)	Q9NWZ3
Discoidin, CUB and LCCL domain-containing protein 2 (DCBLD2)	Q96PD2	Interleukin-5 (IL5)	P05113
DNA fragmentation factor subunit alpha (DFFA)	O00273	Interleukin-6 (IL6)	P05231
Dual adapter for phosphotyrosine and 3-phosphotyrosine and 3-phosphoinositide (DAPP1)	Q9UN19	Interleukin-10 (IL10)	P22301
Dynactin subunit 1 (DCTN1)	Q14203	Interleukin-12 receptor subunit beta-1 (IL12RB1)	P42701
E3 ubiquitin-protein ligase TRIM21 (TRIM21)	P19474	Islet cell autoantigen 1 (ICA1)	Q05084
Egl nine homolog 1 (EGLN1)	Q9GZT9	Keratin, type I cytoskeletal 19 (KRT19)	P08727

Table continues on reverse ▶

Leukocyte immunoglobulin-like receptor subfamily B member 4 (LILRB4)	Q8NHJ6	Protein HEXIM1 (HEXIM1)	O94992
Lymphocyte activation gene 3 protein (LAG3)	P18627	Protein kinase C theta type (PRKCQ)	Q04759
Lymphocyte antigen 75 (LY75)	O60449	Protein sprouty homolog 2 (SPRY2)	O43597
Lysosome-associated membrane glycoprotein 3 (LAMP3)	Q9UQV4	Protein-arginine deiminase type-2 (PADI2)	Q9Y2J8
Mannan-binding lectin serine protease 1 (MASP1)	P48740	SH2 domain-containing protein 1A (SH2D1A)	O60880
Merlin (NF2)	P35240	SH2B adapter protein 3 (SH2B3)	Q9UQQ2
Methylated-DNA--protein-cysteine methyltransferase (MGMT)	P16455	Signaling threshold-regulating transmembrane adapter 1 (SIT1)	Q9Y3P8
Natural cytotoxicity triggering receptor 1 (NCR1)	O76036	SRSF protein kinase 2 (SRPK2)	P78362
Natural killer cells antigen CD94 (KLRD1)	Q13241	Stanniocalcin-1 (STC1)	P52823
Neurabin-2 (PPP1R9B)	Q96SB3	Stromal cell-derived factor 1 (CXCL12)	P48061
Neurotrophin-4 (NTF4)	P34130	T-cell-specific surface glycoprotein CD28 (CD28)	P10747
Nuclear factor of activated T-cells, cytoplasmic 3 (NFATC3)	Q12968	Thioredoxin-dependent peroxide reductase, mitochondrial (PRDX3)	P30048
Parathyroid hormone/parathyroid hormone-related peptide receptor (PTH1R)	Q03431	TNF receptor-associated factor 2 (TRAF2)	Q12933
PC4 and SFRS1-interacting protein (PSIP1)	O75475	TRAF family member-associated NF-kappa-B activator (TANK)	Q92844
Peroxiredoxin-1 (PRDX1)	Q06830	Transcription factor AP-1 (JUN)	P05412
Peroxiredoxin-5, mitochondrial (PRDX5)	P30044	Transcription regulator protein BACH1 (BACH1)	O14867
Phosphoinositide 3-kinase adapter protein 1 (PIK3AP1)	Q6ZUJ8	Triggering receptor expressed on myeloid cells 1 (TREM1)	Q9NP99
Plexin-A4 (PLXNA4)	Q9HCM2	Tripartite motif-containing protein 5 (TRIM5)	Q9C035
Polypeptide N-acetylgalactosaminyltransferase 3 (GALNT3)	Q14435	Tryptase alpha/beta-1 (TPSAB1)	Q15661
Probable ATP-dependent RNA helicase DDX58 (DDX58)	O95786	Tumor necrosis factor receptor superfamily member EDAR (EDAR)	Q9UNE0
Protein FAM3B (FAM3B)	P58499	Zinc finger and BTB domain-containing protein 16 (ZBTB16)	Q05516

For more details visit www.olink.com/immune-response

www.olink.com

For research use only. Not for use in diagnostic procedures.
This product includes a license for non-commercial use. Commercial users may require additional licenses. Please contact Olink Proteomics AB for details.
There are no warranties, expressed or implied, which extend beyond this description. Olink Proteomics AB is not liable for property damage, personal injury, or economic loss caused by this product.
Olink® is a registered trademark of Olink Proteomics AB.

© 2017–2022 Olink Proteomics AB. All third party trademarks are the property of their respective owners.

Olink Proteomics, Dag Hammarskjölds väg 52B , SE-752 37 Uppsala, Sweden

1051, v2.0, 2022-06-14

Olink

Protein assay list

Olink® Target 96 Inflammation

Product number: 95302

Adenosine Deaminase (ADA)	P00813	Fibroblast growth factor 23 (FGF-23)	Q9GZV9
Artemin (ARTN)	Q5T4W7	Fibroblast growth factor 5 (FGF-5)	Q8NF90
Axin-1 (AXIN1)	O15169	Fibroblast growth factor 19 (FGF-19)	O95750
Beta-nerve growth factor (Beta-NGF)	P01138	Fms-related tyrosine kinase 3 ligand (Flt3L)	P49771
Caspase-8 (CASP-8)	Q14790	Fractalkine (CX3CL1)	P78423
C-C motif chemokine 3 (CCL3)	P10147	Glial cell line-derived neurotrophic factor (GDNF)	P39905
C-C motif chemokine 4 (CCL4)	P13236	Hepatocyte growth factor (HGF)	P14210
C-C motif chemokine 19 (CCL19)	Q99731	Interferon gamma (IFN-gamma)	P01579
C-C motif chemokine 20 (CCL20)	P78556	Interleukin-1 alpha (IL-1 alpha)	P01583
C-C motif chemokine 23 (CCL23)	P55773	Interleukin-2 (IL-2)	P60568
C-C motif chemokine 25 (CCL25)	O15444	Interleukin-2 receptor subunit beta (IL-2RB)	P14784
C-C motif chemokine 28 (CCL28)	Q9NRJ3	Interleukin-4 (IL-4)	P05112
CD40L receptor (CD40)	P25942	Interleukin-5 (IL5)	P05113
CUB domain-containing protein 1 (CDCP1)	Q9H5V8	Interleukin-6 (IL6)	P05231
C-X-C motif chemokine 1 (CXCL1)	P09341	Interleukin-7 (IL-7)	P13232
C-X-C motif chemokine 5 (CXCL5)	P42830	Interleukin-8 (IL-8)	P10145
C-X-C motif chemokine 6 (CXCL6)	P80162	Interleukin-10 (IL10)	P22301
C-X-C motif chemokine 9 (CXCL9)	Q07325	Interleukin-10 receptor subunit alpha (IL-10RA)	Q13651
C-X-C motif chemokine 10 (CXCL10)	P02778	Interleukin-10 receptor subunit beta (IL-10RB)	Q08334
C-X-C motif chemokine 11 (CXCL11)	O14625	Interleukin-12 subunit beta (IL-12B)	P29460
Cystatin D (CST5)	P28325	Interleukin-13 (IL-13)	P35225
Delta and Notch-like epidermal growth factor-related receptor (DNER)	Q8NFT8	Interleukin-15 receptor subunit alpha (IL-15RA)	Q13261
Eotaxin (CCL11)	P51671	Interleukin-17A (IL-17A)	Q16552
Eukaryotic translation initiation factor 4E-binding protein 1 (4E-BP1)	Q13541	Interleukin-17C (IL-17C)	Q9P0M4
Fibroblast growth factor 21 (FGF-21)	Q9NSA1	Interleukin-18 (IL-18)	Q14116

Table continues on reverse ▶

Interleukin-18 receptor 1 (IL-18R1)	Q13478	Programmed cell death 1 ligand 1 (PD-L1)	Q9NZQ7
Interleukin-20 (IL-20)	Q9NYY1	Protein S100-A12 (EN-RAGE)	P80511
Interleukin-20 receptor subunit alpha (IL-20RA)	Q9UHF4	Signaling lymphocytic activation molecule (SLAMF1)	Q13291
Interleukin-22 receptor subunit alpha-1 (IL-22 RA1)	Q8N6P7	SIR2-like protein 2 (SIRT2)	Q8IXJ6
Interleukin-24 (IL-24)	Q13007	STAM-binding protein (STAMBP)	O95630
Interleukin-33 (IL-33)	O95760	Stem cell factor (SCF)	P21583
Latency-associated peptide transforming growth factor beta-1 (LAP TGF-beta-1)	P01137	Sulfotransferase 1A1 (ST1A1)	P50225
Leukemia inhibitory factor (LIF)	P15018	T cell surface glycoprotein CD6 isoform (CD6)	Q8WWJ7
Leukemia inhibitory factor receptor (LIF-R)	P42702	T-cell surface glycoprotein CD5 (CD5)	P06127
Macrophage colony-stimulating factor 1 (CSF-1)	P09603	T-cell surface glycoprotein CD8 alpha chain (CD8A)	P01732
Matrix metalloproteinase-1 (MMP-1)	P03956	Thymic stromal lymphopoietin (TSLP)	Q969D9
Matrix metalloproteinase-10 (MMP-10)	P09238	TNF-beta (TNFB)	P01374
Monocyte chemotactic protein 1 (MCP-1)	P13500	TNF-related activation-induced cytokine (TRANCE)	O14788
Monocyte chemotactic protein 2 (MCP-2)	P80075	TNF-related apoptosis-inducing ligand (TRAIL)	P50591
Monocyte chemotactic protein 3 (MCP-3)	P80098	Transforming growth factor alpha (TGF-alpha)	P01135
Monocyte chemotactic protein 4 (MCP-4)	Q99616	Tumor necrosis factor (Ligand) superfamily, member 12 (TWEAK)	O43508
Natural killer cell receptor 2B4 (CD244)	Q9BZW8	Tumor necrosis factor (TNF)	P01375
Neurotrophin-3 (NT-3)	P20783	Tumor necrosis factor ligand superfamily member 14 (TNFSF14)	O43557
Neurturin (NRTN)	Q99748	Tumor necrosis factor receptor superfamily member 9 (TNFRSF9)	Q07011
Oncostatin-M (OSM)	P13725	Urokinase-type plasminogen activator (uPA)	P00749
Osteoprotegerin (OPG)	O00300	Vascular endothelial growth factor A (VEGF-A)	P15692

For more details visit www.olink.com/inflammation

www.olink.com

For research use only. Not for use in diagnostic procedures.
This product includes a license for non-commercial use. Commercial users may require additional licenses. Please contact Olink Proteomics AB for details.
There are no warranties, expressed or implied, which extend beyond this description. Olink Proteomics AB is not liable for property damage, personal injury, or economic loss caused by this product.
Olink® is a registered trademark of Olink Proteomics AB.

© 2017–2022 Olink Proteomics AB. All third party trademarks are the property of their respective owners.

Olink Proteomics, Dag Hammarskjölds väg 52B , SE-752 37 Uppsala, Sweden
1029, v2.0, 2022-06-14

Protein assay list

Olink® Target 96 Metabolism

Product number: 95340

Adenosylhomocysteinase (AHCY)	P23526	CXADR-like membrane protein (CLMP)	Q9H6B4
Adhesion G protein-coupled receptor E2 (ADGRE2)	Q9UHX3	Diablo homolog, mitochondrial (DIABLO)	Q9NR28
Adhesion G-protein coupled receptor G2 (ADGRG2)	Q8IZP9	Dihydropteridine reductase (QDPR)	P09417
Amyloid-like protein 1 (APLP1)	P51693	Dipeptidyl peptidase 2 (DPP7)	Q9UHL4
Angiopoietin-2 (ANGPT2)	O15123	Disabled homolog 2 (DAB2)	P98082
Angiopoietin-related protein 1 (ANGPTL1)	O95841	DNA-(apurinic or apyrimidinic site) lyase (APEX1)	P27695
Angiopoietin-related protein 7 (ANGPTL7)	O43827	Ectonucleoside triphosphate diphosphohydrolase 5 (ENTPD5)	O75356
Annexin A4 (ANXA4)	P09525	Ectonucleotide pyrophosphatase/phosphodiesterase family member 7 (ENPP7)	Q6UWV6
Annexin A11 (ANXA11)	P50995	Eosinophil cationic protein (RNASE3)	P12724
Appetite-regulating hormone (GHRL)	Q9UBU3	Fc receptor-like protein 1 (FCRL1)	Q96LA6
Arginase-1 (ARG1)	P05089	Fructose-1,6-bisphosphatase 1 (FBP1)	P09467
Aromatic-L-amino-acid decarboxylase (DDC)	P20711	Galanin peptides (GAL)	P22466
B-cell antigen receptor complex-associated protein beta chain (CD79B)	P40259	Gamma-enolase (ENO2)	P09104
Cadherin-2 (CDH2)	P19022	Glutaredoxin-1 (GLRX)	P35754
Cadherin-related family member 5 (CDHR5)	Q9HBB8	GRB2-related adapter protein 2 (GRAP2)	O75791
Calsyntenin-2 (CLSTN2)	Q9H4D0	Hepatoma-derived growth factor (HDGF)	P51858
Carbonic anhydrase 13 (CA13)	Q8N1Q1	Inactive tyrosine-protein kinase transmembrane receptor ROR1 (ROR1)	Q01973
Catechol O-methyltransferase (COMT)	P21964	Insulin-like growth factor-binding protein-like 1 (IGFBPL1)	Q8WX77
Cathepsin O (CTSO)	P43234	Integrin beta-7 (ITGB7)	P26010
CD2-associated protein (CD2AP)	Q9Y5K6	Kallikrein-10 (KLK10)	O43240
Chordin-like protein 2 (CHRDL2)	Q6WN34	Kynurenine-oxoglutarate transaminase 1 (KYAT1)	Q16773
Clusterin-like protein 1 (CLUL1)	Q15846	Large proline-rich protein BAG6 (BAG6)	P46379
Coiled-coil domain-containing protein 80 (CCDC80)	Q76M96	Leucine-rich repeats and immunoglobulin-like domains protein 1 (LRIG1)	Q96JA1
Crk-like protein (CRKL)	P46109		
C-type lectin domain family 5 member A (CLEC5A)	Q9NY25	Leukocyte immunoglobulin-like receptor subfamily A member 5 (LILRA5)	A6NI73

Table continues on reverse ▶

Low-density lipoprotein receptor-related protein 11 (LRP11)	Q86VZ4	Scavenger receptor cysteine-rich domain-containing group B protein (SSC4D)	Q8WTU2
Lysophosphatidic acid phosphatase type 6 (ACP6)	Q9NPH0	Sclerostin (SOST)	Q9BQB4
Meprin A subunit beta (MEP1B)	Q16820	Semaphorin-3F (SEMA3F)	Q13275
Meteorin-like protein (METRNL)	Q641Q3	Serpin B6 (SERPINB6)	P35237
Multiple coagulation factor deficiency protein 2 (MCFD2)	Q8NI22	Serpin B8 (SERPINB8)	P50452
NAD kinase (NADK)	O95544	Sialic acid-binding Ig-like lectin 7 (SIGLEC7)	Q9Y286
Nectin-2 (NECTIN2)	Q92692	Sialomucin core protein 24 (CD164)	Q04900
Neural proliferation differentiation and control protein 1 (NPDC1)	Q9NQX5	Soluble calcium-activated nucleotidase 1 (CANT1)	Q8WVQ1
Neuronal pentraxin receptor (NPTXR)	O95502	Sulfatase-modifying factor 2 (SUMF2)	Q8NBJ7
Nodal modulator 1 (NOMO1)	Q15155	Synaptosomal-associated protein 23 (SNAP23)	O00161
N-terminal prohormone brain natriuretic peptide (NT-proBNP)	NA	Syndecan-4 (SDC4)	P31431
Paired immunoglobulin-like type 2 receptor beta (PILRB)	Q9UKJ0	T-cell surface glycoprotein CD1c (CD1C)	P29017
Peptidyl-prolyl cis-trans isomerase FKBP4 (FKBP4)	Q02790	Thimet oligopeptidase (THOP1)	P52888
Phosphoprotein associated with glycosphingolipid-enriched microdomains 1 (PAG1)	Q9NWQ8	Thioredoxin domain-containing protein 5 (TXNDC5)	Q8NBS9
Pro-cathepsin H (CTSH)	P09668	Thymidine phosphorylase (TYMP)	P19971
Protein FAM3C (FAM3C)	Q92520	Thyrotropin subunit beta (TSHB)	P01222
Protein phosphatase inhibitor 2 (PPP1R2)	P41236	Trefoil factor 2 (TFF2)	Q03403
Protein S100-P (S100P)	P25815	Tubulointerstitial nephritis antigen-like (TINAGL1)	Q9GZM7
Regenerating islet-derived protein 4 (REG4)	Q9BYZ8	Tyrosine-protein kinase receptor TYRO3 (TYRO3)	Q06418
Reticulon-4 receptor (RTN4R)	Q9BZR6	Ubiquitin carboxyl-terminal hydrolase 8 (USP8)	P40818
Retinal dehydrogenase 1 (ALDH1A1)	P00352	Versican core protein (VCAN)	P13611
Ribosyldihydronicotinamide dehydrogenase [quinone] (NQO2)	P16083		

For more details visit www.olink.com/metabolism

www.olink.com

For research use only. Not for use in diagnostic procedures.
This product includes a license for non-commercial use. Commercial users may require additional licenses. Please contact Olink Proteomics AB for details.
There are no warranties, expressed or implied, which extend beyond this description. Olink Proteomics AB is not liable for property damage, personal injury, or economic loss caused by this product.
Olink® is a registered trademark of Olink Proteomics AB.

© 2017–2022 Olink Proteomics AB. All third party trademarks are the property of their respective owners.

Olink Proteomics, Dag Hammarskjölds väg 52B , SE-752 37 Uppsala, Sweden

1053, v2.0, 2022-06-14

Olink®

Protein assay list

Olink® Target 96 Neuro Exploratory

Product number: 95391

6-pyruvoyl tetrahydrobiopterin synthase (PTS)	Q03393
Adhesion G protein-coupled receptor B3 (ADGRB3)	O60242
Alanyl-tRNA editing protein Aarsd1 (AARSD1)	Q9BTE6
Alpha-(1,6)-fucosyltransferase (FUT8)	Q9BYC5
Amiloride-sensitive amine oxidase [copper-containing] (AOC1)	P19801
Annexin A10 (ANXA10)	Q9UJ72
Asialoglycoprotein receptor 1 (ASGR1)	P07306
Beta-defensin 4A (DEFB4A)	O15263
Beta-klotho (KLB)	Q86Z14
Bis(5'-adenosyl)-triphosphatase (FHIT)	P49789
Bone marrow stromal antigen 2 (BST2)	Q10589
Cadherin-15 (CDH15)	P55291
Cadherin-17 (CDH17)	Q12864
Calcineurin subunit B type 1 (PPP3R1)	P63098
Calcium-regulated heat-stable protein 1 (CARHSP1)	Q9Y2V2
Calsyntenin-1 (CLSTN1)	O94985
Carcinoembryonic antigen-related cell adhesion molecule 3 (CEACAM3)	P40198
Cardiotrophin-1 (CTF1)	Q16619
C-C motif chemokine 27 (CCL27)	Q9Y4X3
CD302 antigen (CD302)	Q8IX05
CD63 antigen (CD63)	P08962
Centrin-2 (CETN2)	P41208
Ceramide transfer protein (CERT1)	Q9Y5P4
Cysteine-rich protein 2 (CRIP2)	P52943
Death domain-containing protein CRADD (CRADD)	P78560
Desmoglein-3 (DSG3)	P32926
Dipeptidase 1 (DPEP1)	P16444
Dipeptidase 2 (DPEP2)	Q9H4A9
Disintegrin and metalloproteinase domain-containing protein 15 (ADAM15)	Q13444
Dual specificity protein phosphatase 3 (DUSP3)	P51452
E3 ubiquitin-protein ligase RNF31 (RNF31)	Q96EP0
Endoplasmin (HSP90B1)	P14625
Endothelin-converting enzyme 1 (ECE1)	P42892
Ephrin type-A receptor 10 (EPHA10)	Q5JZY3
Eukaryotic translation initiation factor 4B (EIF4B)	P23588
Fibroblast growth factor receptor 2 (FGFR2)	P21802
Gamma-interferon-inducible lysosomal thiol reductase (IFI30)	P13284
Gamma-synuclein (SNCG)	O76070
Glutathione hydrolase 5 proenzyme (GGT5)	P36269
Glutathione S-transferase P (GSTP1)	P09211
Glycodelin (PAEP)	P09466
Group 10 secretory phospholipase A2 (PLA2G10)	O15496
Guanylate-binding protein 2 (GBP2)	P32456
Heme oxygenase 2 (HMOX2)	P30519
Immunoglobulin alpha Fc receptor (FCAR)	P24071
Immunoglobulin superfamily containing leucine-rich repeat protein 2 (ISLR2)	Q6UXK2
Inhibitor of growth protein 1 (ING1)	Q9UK53
Inositol monophosphatase 1 (IMPA1)	P29218
Integrin-linked kinase-associated serine/threonine phosphatase 2C (ILKAP)	Q9H0C8
Interferon lambda-1 (IFNL1)	Q8IU54
Interleukin-15 (IL15)	P40933

Table continues on reverse ▶

Interleukin-3 receptor subunit alpha (IL3RA)	P26951
Interleukin-32 (IL32)	P24001
KIF1-binding protein (KIF1BP)	Q96EK5
Killer cell immunoglobulin-like receptor 2DL3 (KIR2DL3)	P43628
Kin of IRRE-like protein 2 (KIRREL2)	Q6UWL6
Latent-transforming growth factor beta-binding protein 3 (LTBP3)	Q9NS15
Leptin receptor (LEPR)	P48357
Mitotic spindle assembly checkpoint protein MAD1 (MAD1L1)	Q9Y6D9
Myeloid cell surface antigen CD33 (CD33)	P20138
N-alpha-acetyltransferase 10 (NAA10)	P41227
NEDD4-like E3 ubiquitin-protein ligase WWP2 (WWP2)	O00308
NEDD8-conjugating enzyme UBE2F (UBE2F)	Q969M7
Neurexophilin-1 (NXPH1)	P58417
Neurofilament light polypeptide (NEFL)	P07196
Nucleophosmin (NPM1)	P06748
Peptidyl-prolyl cis-trans isomerase FKBP5 (FKBP5)	Q13451
Peptidyl-prolyl cis-trans isomerase FKBP7 (FKBP7)	Q9Y680
Phosphoethanolamine/phosphocholine phosphatase (PHOSPHO1)	Q8TCT1
Phosphomevalonate kinase (PMVK)	Q15126
Phosphoribosyltransferase domain-containing protein 1 (PRTFDC1)	Q9NRG1
Prefoldin subunit 2 (PFDN2)	Q9UHV9
Pregnancy-specific beta-1-glycoprotein 1 (PSG1)	P11464
Proepiregulin (EREG)	O14944
Proline-rich AKT1 substrate 1 (AKT1S1)	Q96B36
Proteasome activator complex subunit 1 (PSME1)	Q06323
Protein ABHD14B (ABHD14B)	Q96IU4
Protein NDRG1 (NDRG1)	Q92597
Ribokinase (RBKS)	Q9H477
Ribosomal protein S6 kinase beta-1 (RPS6KB1)	P23443
Secreted frizzled-related protein 1 (SFRP1)	Q8N474
Signal recognition particle 14 kDa protein (SRP14)	P37108
SPARC-related modular calcium-binding protein 1 (SMOC1)	Q9H4F8
Teratocarcinoma-derived growth factor 1 (TDGF1)	P13385
Transmembrane glycoprotein NMB (GPNMB)	Q14956
Tubulin polymerization-promoting protein family member 3 (TPPP3)	Q9BW30
Tubulin-folding cofactor B (TBCB)	Q99426
Tumor necrosis factor receptor superfamily member 13C (TNFRSF13C)	Q96RJ3
Tyrosine-protein phosphatase non-receptor type 1 (PTPN1)	P18031
V-set and transmembrane domain-containing protein 1 (VSTM1)	Q6UX27
V-type proton ATPase subunit F (ATP6V1F)	Q16864
Zinc finger protein Helios (IKZF2)	Q9UKS7

For more details visit www.olink.com/neuro-exploratory

www.olink.com

For research use only. Not for use in diagnostic procedures.
This product includes a license for non-commercial use. Commercial users may require additional licenses. Please contact Olink Proteomics AB for details.
There are no warranties, expressed or implied, which extend beyond this description. Olink Proteomics AB is not liable for property damage, personal injury, or economic loss caused by this product.
Olink® is a registered trademark of Olink Proteomics AB.

© 2017–2022 Olink Proteomics AB. All third party trademarks are the property of their respective owners.
Olink Proteomics, Dag Hammarskjölds väg 52B , SE-752 37 Uppsala, Sweden
1100, v2.0, 2022-08-16

Olink®

Protein assay list

Olink® Target 96 Neurology

Product number: 95801

ADP-ribosyl cyclase/cyclic ADP-ribose hydrolase 1 (CD38)	P28907
Alpha-2-macroglobulin receptor-associated protein (Alpha-2-MRAP)	P30533
BDNF/NT-3 growth factors receptor (NTRK2)	Q16620
Beta-nerve growth factor (Beta-NGF)	P01138
Bone morphogenetic protein 4 (BMP-4)	P12644
Brevican core protein (BCAN)	Q96GW7
Brorin (VWC2)	Q2TAL6
Cadherin-3 (CDH3)	P22223
Cadherin-6 (CDH6)	P55285
Carboxypeptidase A2 (CPA2)	P48052
Carboxypeptidase M (CPM)	P14384
Cathepsin S (CTSS)	P25774
Cell adhesion molecule 3 (CADM3)	Q8N126
Cell surface glycoprotein CD200 receptor 1 (CD200R1)	Q8TD46
CMRF35-like molecule 1 (CLM-1)	Q8TDQ1
CMRF35-like molecule 6 (CLM-6)	Q08708
Contactin-5 (CNTN5)	O94779
C-type lectin domain family 1 member B (CLEC1B)	Q9P126
C-type lectin domain family 10 member A (CLEC10A)	Q8IUN9
Cytotoxic and regulatory T-cell molecule (CRTAM)	O95727
Dickkopf-related protein 4 (Dkk-4)	Q9UBT3
Dipeptidyl peptidase 1 (CTSC)	P53634
Disintegrin and metalloproteinase domain-containing protein 22 (ADAM 22)	Q9P0K1
Disintegrin and metalloproteinase domain-containing protein 23 (ADAM 23)	O75077
Draxin (DRAXIN)	Q8NBI3
Ephrin type-B receptor 6 (EPHB6)	O15197
Ephrin-A4 (EFNA4)	P52798
Epithelial discoidin domain-containing receptor 1 (DDR1)	Q08345
Ezrin (EZR)	P15311
Fc receptor-like protein 2 (FcRL2)	Q96LA5
Galectin-8 (gal-8)	O00214
GDNF family receptor alpha-1 (GFR-alpha-1)	P56159
GDNF family receptor alpha-3 (GDNFR-alpha-3)	O60609
Glial cell line-derived neurotrophic factor (GDNF)	P39905
Glypican-5 (GCP5)	P78333
Granulocyte Colony-Stimulating Factor (G-CSF)	P09919
Granulocyte-macrophage colony-stimulating factor receptor subunit alpha (GM-CSF-R-alpha)	P15509
Granzyme A (GZMA)	P12544
Growth/differentiation factor 8 (GDF-8)	O14793
Hydroxyacylglutathione hydrolase, mitochondrial (HAGH)	Q16775
Interleukin-5 receptor subunit alpha (IL-5R-alpha)	Q01344
Interleukin-12 (IL-12)	P29460, P29459
Junctional adhesion molecule B (JAM-B)	P57087
Kynureninase (KYNU)	Q16719
Latexin (LXN)	Q9BS40
Layilin (LAYN)	Q6UX15
Leucine-rich repeat transmembrane protein FLRT2 (FLRT2)	O43155
Leukocyte-associated immunoglobulin-like receptor 2 (LAIR-2)	Q6ISS4
Linker for activation of T-cells family member 1 (LAT)	O43561
Lysosome membrane protein 2 (SCARB2)	Q14108

Table continues on reverse ▶

Macrophage scavenger receptor types I and II (MSR1)	P21757	Protogenin (PRTG)	Q2VWP7
MAM domain-containing glycosylphosphatidylinositol anchor protein 1 (MDGA1)	Q8NFP4	Repulsive guidance molecule A (RGMA)	Q96B86
Matrilin-3 (MATN3)	O15232	RGM domain family member B (RGMB)	Q6NW40
Mesencephalic astrocyte-derived neurotrophic factor (MANF)	P55145	Roundabout homolog 2 (ROBO2)	Q9HCK4
Microtubule-associated protein tau (MAPT)	P10636	R-spondin-1 (RSPO1)	Q2MKA7
N-acylethanolamine-hydrolyzing acid amidase (NAAA)	Q02083	Scavenger receptor class A member 5 (SCARA5)	Q6ZMJ2
Neprilysin (NEP)	P08473	Scavenger receptor class F member 2 (SCARF2)	Q96GP6
Netrin receptor UNC5C (UNC5C)	O95185	Secreted frizzled-related protein 3 (sFRP-3)	Q92765
Neuroblastoma suppressor of tumorigenicity 1 (NBL1)	P41271	Serine/threonine-protein kinase receptor R3 (SKR3)	P37023
Neurocan core protein (NCAN)	O14594	Sialic acid-binding Ig-like lectin 9 (Siglec-9)	Q9Y336
Neuronal cell adhesion molecule (Nr-CAM)	Q92823	Sialoadhesin (SIGLEC1)	Q9BZZ2
Neuropilin-2 (NRP2)	O60462	SPARC-related modular calcium-binding protein 2 (SMOC2)	Q9H3U7
Neutral ceramidase (N-CDase)	Q9NR71	Sphingomyelin phosphodiesterase (SMPD1)	P17405
Nicotinamide/nicotinic acid mononucleotide adenylyltransferase 1 (NMNAT1)	Q9HAN9	Tenascin-R (TN-R)	Q92752
NKG2D ligand 2 (N2DL-2)	Q9BZM5	Testican-1 (SPOCK1)	Q08629
NT-3 growth factor receptor (NTRK3)	Q16288	Thy-1 membrane glycoprotein (THY 1)	P04216
OX-2 membrane glycoprotein (CD200)	P41217	Transmembrane protease serine 5 (TMPRSS5)	Q9H3S3
Platelet-derived growth factor receptor alpha (PDGF-R-alpha)	P16234	Tumor necrosis factor receptor superfamily member 12A (TNFRSF12A)	Q9NP84
Plexin-B1 (PLXNB1)	O43157	Tumor necrosis factor receptor superfamily member 21 (TNFRSF21)	O75509
Plexin-B3 (PLXNB3)	Q9ULL4	Tumor necrosis factor receptor superfamily member 27 (EDA2R)	Q9HAV5
Poliovirus receptor (PVR)	P15151	WAP, Kazal, immunoglobulin, Kunitz and NTR domain-containing protein 1 (WFIKKN1)	Q96NZ8

For more details visit www.olink.com/neurology

www.olink.com

For research use only. Not for use in diagnostic procedures.
This product includes a license for non-commercial use. Commercial users may require additional licenses. Please contact Olink Proteomics AB for details.
There are no warranties, expressed or implied, which extend beyond this description. Olink Proteomics AB is not liable for property damage, personal injury, or economic loss caused by this product.
Olink® is a registered trademark of Olink Proteomics AB.

© 2017–2022 Olink Proteomics AB. All third party trademarks are the property of their respective owners.

Olink Proteomics, Dag Hammarskjölds väg 52B , SE-752 37 Uppsala, Sweden
1035, v2.0, 2022-06-16

Olink®

Protein assay list

Olink® Target 96 Oncology II

Product number: 95700

5'-nucleotidase (5'-NT)	P21589	Fc receptor-like B (FCRLB)	Q6BAA4
A disintegrin and metalloproteinase with thrombospondin motifs 15 (ADAM-TS 15)	Q8TE58	Fibroblast growth factor-binding protein 1 (FGF-BP1)	Q14512
Alpha-taxilin (TXLNA)	P40222	Folate receptor alpha (FR-alpha)	P15328
Amphiregulin (AREG)	P15514	Folate receptor gamma (FR-gamma)	P41439
Annexin A1 (ANXA1)	P04083	Furin (FUR)	P09958
Carbonic anhydrase IX (CAIX)	Q16790	Galectin-1 (Gal-1)	P09382
Carboxypeptidase E (CPE)	P16870	Glypican-1 (GPC1)	P35052
Carcinoembryonic antigen (CEA)	P06731	Granzyme B (GZMB)	P10144
Carcinoembryonic antigen-related cell adhesion molecule 1 (CEACAM1)	P13688	Granzyme H (GZMH)	P20718
Cathepsin L2 (CTSV)	O60911	Hepatocyte growth factor (HGF)	P14210
CD27 antigen (CD27)	P26842	ICOS ligand (ICOSLG)	O75144
CD160 antigen (CD160)	O95971	Insulin-like growth factor 1 receptor (IGF1R)	P08069
CD48 antigen (CD48)	P09326	Integrin alpha-V (ITGAV)	P06756
CD70 antigen (CD70)	P32970	Integrin beta-5 (ITGB5)	P18084
Cornulin (CRNN)	Q9UBG3	Interferon gamma receptor 1 (IFN-gamma-R1)	P15260
C-type lectin domain family 4 member K (CD207)	Q9UJ71	Interleukin-6 (IL6)	P05231
C-X-C motif chemokine 13 (CXCL13)	O43927	Kallikrein-8 (hK8)	O60259
Cyclin-dependent kinase inhibitor 1 (CDKN1A)	P38936	Kallikrein-11 (hK11)	Q9UBX7
Delta-like protein 1 (DLL1)	O00548	Kallikrein-13 (KLK13)	Q9UKR3
Disintegrin and metalloproteinase domain-containing protein 8 (ADAM8)	P78325	Kallikrein-14 (hK14)	Q9P0G3
		Ly6/PLAUR domain-containing protein 3 (LYPD3)	O95274
Endothelial cell-specific molecule 1 (ESM-1)	Q9NQ30	Melanoma-derived growth regulatory protein (MIA)	Q16674
Ephrin type-A receptor 2 (EPHA2)	P29317	Mesothelin (MSLN)	Q13421
Fas antigen ligand (FasL)	P48023	Methionine aminopeptidase 2 (MetAP 2)	P50579
FAS-associated death domain protein (FADD)	Q13158	MHC class I polypeptide-related sequence A/B (MIC-A/B)	Q29983, Q29980

Table continues on reverse ▶

Midkine (MK)	P21741	Tissue factor pathway inhibitor 2 (TFPI-2)	P48307
Mothers against decapentaplegic homolog 5 (MAD homolog 5)	Q99717	T-lymphocyte surface antigen Ly-9 (LY9)	Q9HBG7
Mucin-16 (MUC-16)	Q8WXI7	TNF-related apoptosis-inducing ligand (TRAIL)	P50591
Nectin-4 (PVRL4)	Q96NY8	Toll-like receptor 3 (TLR3)	O15455
Pancreatic prohormone (PPY)	P01298	Transforming growth factor alpha (TGF-alpha)	P01135
Podocalyxin (PODXL)	O00592	Transmembrane glycoprotein NMB (GPNMB)	Q14956
Pro-epidermal growth factor (EGF)	P01133	Tumor necrosis factor ligand superfamily member 13 (TNFSF13)	O75888
Protein CYR61 (CYR61)	O00622	Tumor necrosis factor receptor superfamily member 4 (TNFRSF4)	P43489
Protein S100-A11 (S100A11)	P31949	Tumor necrosis factor receptor superfamily member 6B (TNFRSF6B)	O95407
Protein S100-A4 (S100A4)	P26447	Tumor necrosis factor receptor superfamily member 19 (TNFRSF19)	Q9NS68
Proto-oncogene tyrosine-protein kinase receptor Ret (RET)	P07949	Tyrosine-protein kinase ABL1 (ABL1)	P00519
Receptor tyrosine-protein kinase erbB-2 (ErbB2/HER2)	P04626	Tyrosine-protein kinase Lyn (LYN)	P07948
Receptor tyrosine-protein kinase erbB-3 (ErbB3/HER3)	P21860	WAP four-disulfide core domain protein 2 (WFDC2)	Q14508
Receptor tyrosine-protein kinase erbB-4 (ErbB4/HER4)	Q15303	Vascular endothelial growth factor A (VEGF-A)	P15692
R-spondin-3 (RSPO3)	Q9BXY4	Vascular endothelial growth factor receptor 2 (VEGFR-2)	P35968
Secretory carrier-associated membrane protein 3 (SCAMP3)	O14828	Vascular endothelial growth factor receptor 3 (VEGFR-3)	P35916
Seizure 6-like protein (SEZ6L)	Q9BYH1	VEGF-co regulated chemokine 1 (CXL17)	Q6UXB2
SPARC (SPARC)	P09486	Vimentin (VIM)	P08670
Stem cell factor (SCF)	P21583	Wnt inhibitory factor 1 (WIF-1)	Q9Y5W5
Syndecan-1 (SYND1)	P18827	WNT1-inducible-signaling pathway protein 1 (WISP-1)	O95388
T-cell leukemia / lymphoma protein 1A (TCL1A)	P56279	Xaa-Pro aminopeptidase 2 (XPNPEP2)	O43895
TGF-beta receptor type-2 (TGFR-2)	P37173		

For more details visit www.olink.com/onc2

www.olink.com

For research use only. Not for use in diagnostic procedures.
This product includes a license for non-commercial use. Commercial users may require additional licenses. Please contact Olink Proteomics AB for details.
There are no warranties, expressed or implied, which extend beyond this description. Olink Proteomics AB is not liable for property damage, personal injury, or economic loss caused by this product.
Olink® is a registered trademark of Olink Proteomics AB.

© 2017–2022 Olink Proteomics AB. All third party trademarks are the property of their respective owners.

Olink Proteomics, Dag Hammarskjölds väg 52B , SE-752 37 Uppsala, Sweden
1032, v2.0, 2022-06-14

Olink®

Protein assay list

Olink® Target 96 Organ Damage

Product number: 95331

5'-AMP-activated protein kinase subunit beta-1 (PRKAB1)	Q9Y478	Enteropeptidase (TMPRSS15)	P98073
Adhesion G-protein coupled receptor G1 (ADGRG1)	Q9Y653	Epidermal growth factor-like protein 7 (EGFL7)	Q9UHF1
Aldehyde dehydrogenase, dimeric NADP-preferring (ALDH3A1)	P30838	Erbin (ERBIN)	Q96RT1
Anterior gradient protein 2 homolog (AGR2)	O95994	Erythropoietin (EPO)	P01588
Apoptosis-inducing factor 1, mitochondrial (AIFM1)	O95831	Fatty acid-binding protein 9 (FABP9)	Q0Z7S8
B-cell scaffold protein with ankyrin repeats (BANK1)	Q8NDB2	Forkhead box protein O1 (FOXO1)	Q12778
BH3-interacting domain death agonist (BID)	P55957	Fructose-2,6-bisphosphatase TIGAR (TIGAR)	Q9NQ88
BMP and activin membrane-bound inhibitor homolog (BAMBI)	Q13145	Hematopoietic prostaglandin D synthase (HPGDS)	O60760
		Inactive tyrosine-protein kinase 7 (PTK7)	Q13308
Calcitonin (CALCA)	P01258	Integrin beta-1-binding protein 1 (ITGB1BP1)	O14713
Calreticulin (CALR)	P27797	Interferon-inducible double-stranded RNA-dependent protein kinase activator A (PRKRA)	O75569
Carbonic anhydrase 12 (CA12)	O43570		
Carbonic anhydrase 14 (CA14)	Q9ULX7	Kidney Injury Molecule (KIM1)	Q96D42
Casein kinase I isoform delta (CSNK1D)	P48730	Killer cell immunoglobulin-like receptor 3DL1 (KIR3DL1)	P43629
Claspin (CLSPN)	Q9HAW4	Leukotriene A-4 hydrolase (LTA4H)	P09960
CMP-N-acetylneuraminate-beta-galactosamide-alpha-2,3-sialyltransferase 1 (ST3GAL1)	Q11201	Linker for activation of T-cells family member 2 (LAT2)	Q9GZY6
		Lutropin subunit beta (LHB)	P01229
Cocaine esterase (CES2)	O00748	Macrophage erythroblast attacher (MAEA)	Q7L5Y9
Contactin-2 (CNTN2)	Q02246	Macrophage-capping protein (CAPG)	P40121
Corticoliberin (CRH)	P06850		
C-type lectin domain family 1 member A (CLEC1A)	Q8NC01	Melanoma-associated antigen D1 (MAGED1)	Q9Y5V3
C-type natriuretic peptide (NPPC)	P23582	Methionine aminopeptidase 1 (METAP1)	P53582
Desmoglein-4 (DSG4)	Q86SJ6	Mevalonate kinase (MVK)	Q03426
Dipeptidyl aminopeptidase-like protein 6 (DPP6)	P42658	Mitogen-activated protein kinase kinase kinase kinase 5 (MAP4K5)	Q9Y4K4
DNA topoisomerase 2-beta (TOP2B)	Q02880	Mothers against decapentaplegic homolog 1 (SMAD1)	Q15797
Ectonucleoside triphosphate diphosphohydrolase 2 (ENTPD2)	Q9Y5L3	NAD-dependent protein deacylase sirtuin-5, mitochondrial (SIRT5)	Q9NXA8
Ectonucleoside triphosphate diphosphohydrolase 6 (ENTPD6)	O75354	NEDD8 ultimate buster 1 (NUB1)	Q9Y5A7
EGF-like repeat and discoidin I-like domain-containing protein 3 (EDIL3)	O43854	Neutrophil cytosol factor 2 (NCF2)	P19878

Table continues on reverse ▶

Nibrin (NBN)	O60934	Protein max (MAX)	P61244
Nitric oxide synthase, endothelial (NOS3)	P29474	Protein phosphatase 1B (PPM1B)	O75688
Nucleobindin-2 (NUCB2)	P80303	[Pyruvate dehydrogenase [acetyl-transferring]]-phosphatase 1, mitochondrial (PDP1)	Q9P0J1
Parvalbumin alpha (PVALB)	P20472	Ras association domain-containing protein 2 (RASSF2)	P50749
Paxillin (PXN)	P49023	Ras GTPase-activating protein 1 (RASA1)	P20936
Peptidyl-prolyl cis-trans isomerase FKBP1B (FKBP1B)	P68106	Receptor-type tyrosine-protein phosphatase eta (PTPRJ)	Q12913
Perilipin-1 (PLIN1)	O60240	Renin receptor (ATP6AP2)	O75787
Phosphatidylinositol 3,4,5-trisphosphate 5-phosphatase 2 (INPPL1)	O15357	REST corepressor 1 (RCOR1)	Q9UKL0
Placenta growth factor (PGF)	P49763	Retinoic acid receptor responder protein 1 (RARRES1)	P49788
Platelet-derived growth factor C (PDGFC)	Q9NRA1	Ribonucleoside-diphosphate reductase subunit M2 B (RRM2B)	Q7LG56
Pleiotrophin (PTN)	P21246	Serpin A9 (SERPINA9)	Q86WD7
Plexin domain-containing protein 1 (PLXDC1)	Q8IUK5	Serum paraoxonase/arylesterase 2 (PON2)	Q15165
Polypeptide N-acetylgalactosaminyltransferase 10 (GALNT10)	Q86SR1	Syntaxin-8 (STX8)	Q9UNK0
Probetacellulin (BTC)	P35070	Syntaxin-binding protein 3 (STXBP3)	O00186
Programmed cell death protein 1 (PDCD1)	Q15116	Troponin I, cardiac muscle (TNNI3)	P19429
Prolow-density lipoprotein receptor-related protein 1 (LRP1)	Q07954	Tyrosine-protein kinase Fes/Fps (FES)	P07332
Proteasome subunit alpha type-1 (PSMA1)	P25786	Tyrosine-protein kinase Fgr (FGR)	P09769
Protein amnionless (AMN)	Q9BXJ7	Tyrosine-protein kinase Yes (YES1)	P07947
Protein enabled homolog (ENAH)	Q8N8S7	Vasohibin-1 (VASH1)	Q7L8A9
Protein fosB (FOSB)	P53539	Wiskott-Aldrich syndrome protein (WAS)	P42768

For more details visit www.olink.com/organ-damage

www.olink.com

For research use only. Not for use in diagnostic procedures.
This product includes a license for non-commercial use. Commercial users may require additional licenses. Please contact Olink Proteomics AB for details.
There are no warranties, expressed or implied, which extend beyond this description. Olink Proteomics AB is not liable for property damage, personal injury, or economic loss caused by this product.
Olink® is a registered trademark of Olink Proteomics AB.

© 2017–2022 Olink Proteomics AB. All third party trademarks are the property of their respective owners.

Olink Proteomics, Dag Hammarskjölds väg 52B , SE-752 37 Uppsala, Sweden

1052, v2.0, 2022-06-14
