# Supplementary material for: Cohort profile: The Belgian I AM frontier prospective cohort study for comprehensive health outcome exploration
Source: PLoS One. 2025 Jun 12;20(6):e0326024. doi: 10.1371/journal.pone.0326024 (PMC12161581; doi:10.1371/journal.pone.0326024)
Supplement: S3 Table — (PDF) [file pone.0326024.s007.pdf]

LB-0428 11/2019

Nightingale Blood Biomarker Analysis Service

Boost your research with comprehensive blood metabolite insights.

Powerful platform enabled by NMR

Unique combination of biomarkers

Biomarkers include clinically validated routine markers, emerging biomarkers with strong medical relevance and promising, new biomarkers.

Robust and highly reproducible results

Our fully automated analysis process is constantly monitored under a certified quality management system. And the NMR technology allows high reproducibility which ensures consistent and reliable results across all sample sets.

Fast, cost-efficient and scalable technology

We use a high-throughput NMR technology which ensures efficient analysis for sample sets of all sizes without batch effects.

Accurate and fully quantified metabolic data

Not only our analysis, but even our quantification process of the NMR spectral data is fully automated, which provides precise and accurate metabolite results in absolute concentration units.

Comprehensive overview of an individual's health

Biomarkers in our panel provide a physiologically meaningful picture of the overall health making it possible to explore novel connections between metabolites and an individual's health status.

Certified quality management system

Nightingale's quality management system has been certified according to EN ISO 13485 standard.

Tech specifications

**Technology**	^{1}H NMR Spectroscopy, Nightingale Health's proprietary analysis
**Specimen type**	Serum and Plasma
**Sample volume**	100 µL and 350 µL
**Number of biomarkers**	250
**Result units**	Absolute biomarker quantification (mmol/l or g/l)
**Sample storage**	Long-term storage -70˚C or below

Clinically validated biomarkers

**Cholesterol**	
Total cholesterol	mmol/l
VLDL cholesterol	mmol/l
Clinical LDL cholesterol	mmol/l
HDL cholesterol	mmol/l
**Triglycerides**	
Total triglycerides	mmol/l
**Apolipoproteins**	
Apolipoprotein B	g/l
Apolipoprotein A1	g/l
Ratio of apolipoprotein B to apolipoprotein A1	ratio
**Fatty acids**	
Total fatty acids	mmol/l
Omega-3 fatty acids	mmol/l
Omega-6 fatty acids	mmol/l
Monounsaturated fatty acids	mmol/l
Saturated fatty acids	mmol/l
**Fatty acid ratios**	
Ratio of omega-3 fatty acids to total fatty acids	%
Ratio of omega-6 fatty acids to total fatty acids	%
Ratio of monounsaturated fatty acids to total fatty acids	%
Ratio of saturated fatty acids to total fatty acids	%
**Branched-chain amino acids**	
Isoleucine	mmol/l
Leucine	mmol/l
Valine	mmol/l
**Glycolysis related metabolites**	
Glucose	mmol/l
**Fluid balance**	
Creatinine	mmol/l
**Inflammation**	
Glycoprotein acetyls	mmol/l

List of all biomarkers

Metabolite	Unit
**Cholesterol**	
Total cholesterol	mmol/l
Total cholesterol minus HDL-C	mmol/l
Remnant cholesterol (non-HDL, non-LDL -cholesterol)	mmol/l
VLDL cholesterol	mmol/l
Clinical LDL cholesterol	mmol/l
LDL cholesterol	mmol/l
HDL cholesterol	mmol/l
**Triglycerides**	
Total triglycerides	mmol/l
Triglycerides in VLDL	mmol/l
Triglycerides in LDL	mmol/l
Triglycerides in HDL	mmol/l
**Phospholipids**	
Total phospholipids in lipoprotein particles	mmol/l
Phospholipids in VLDL	mmol/l
Phospholipids in LDL	mmol/l
Phospholipids in HDL	mmol/l
**Cholesteryl esters**	
Total esterified cholesterol	mmol/l
Cholesterol esters in VLDL	mmol/l
Cholesterol esters in LDL	mmol/l
Cholesterol esters in HDL	mmol/l
**Free cholesterol**	
Total free cholesterol	mmol/l
Free cholesterol in VLDL	mmol/l
Free cholesterol in LDL	mmol/l
Free cholesterol in HDL	mmol/l
**Total lipids**	
Total lipids in lipoprotein particles	mmol/l
Total lipids in VLDL	mmol/l
Total lipids in LDL	mmol/l
Total lipids in HDL	mmol/l
**Lipoprotein particle concentrations**	
Total concentration of lipoprotein particles	mmol/l
Concentration of VLDL particles	mmol/l
Concentration of LDL particles	mmol/l
Concentration of HDL particles	mmol/l
**Lipoprotein particle sizes**	
Average diameter for VLDL particles	nm
Average diameter for LDL particles	nm
Average diameter for HDL particles	nm

Metabolite	Unit
**Other lipids**	
Phosphoglycerides	mmol/l
Ratio of triglycerides to phosphoglycerides	ratio
Total cholines	mmol/l
Phosphatidylcholines	mmol/l
Sphingomyelins	mmol/l
**Apolipoproteins**	
Apolipoprotein B	g/l
Apolipoprotein A1	g/l
Ratio of apolipoprotein B to apolipoprotein A1	ratio
**Fatty acids**	
Total fatty acids	mmol/l
Degree of unsaturation	degree
Omega-3 fatty acids	mmol/l
Omega-6 fatty acids	mmol/l
Polyunsaturated fatty acids	mmol/l
Monounsaturated fatty acids	mmol/l
Saturated fatty acids	mmol/l
Linoleic acid	mmol/l
Docosahexaenoic acid	mmol/l
**Fatty acid ratios**	
Ratio of omega-3 fatty acids to total fatty acids	%
Ratio of omega-6 fatty acids to total fatty acids	%
Ratio of polyunsaturated fatty acids to total fatty acids	%
Ratio of monounsaturated fatty acids to total fatty acids	%
Ratio of saturated fatty acids to total fatty acids	%
Ratio of linoleic acid to total fatty acids	%
Ratio of docosahexaenoic acid to total fatty acids	%
Ratio of polyunsaturated fatty acids to monounsaturated fatty acids	ratio
Ratio of omega-6 fatty acids to omega-3 fatty acids	ratio
**Amino acids**	
Alanine	mmol/l
Glutamine	mmol/l
Glycine	mmol/l
Histidine	mmol/l
**Branched-chain amino acids**	
Total concentration of branded -chain amino acids (leucine + isoleucine + valine)	mmol/l
Isoleucine	mmol/l
Leucine	mmol/l
Valine	mmol/l
**Aromatic amino acids**	
Phenylalanine	mmol/l
Tyrosine	mmol/l

Metabolite	Unit
**Glycolysis related metabolites**	
Glucose	mmol/l
Lactate	mmol/l
Pyruvate	mmol/l
Citrate **	mmol/l
Glycerol *	mmol/l
**Ketone bodies**	
3-Hydroxybutyrate	mmol/l
Acetate	mmol/l
Acetoacetate	mmol/l
Acetone	mmol/l
**Fluid balance**	
Creatinine	mmol/l
Albumin	g/l
**Inflammation**	
Glycoprotein acetyls	mmol/l
**Lipoprotein subclasses**	
**Chylomicrons and extremely large VLDL (particle diameters from 75 nm upwards)**	
Concentration of chylomicrons and extremely large VLDL particles	mmol/l
Total lipids in chylomicrons and extremely large VLDL	mmol/l
Phospholipids in chylomicrons and extremely large VLDL	mmol/l
Cholesterol in chylomicrons and extremely large VLDL	mmol/l
Cholesteryl esters in chylomicrons and extremely large VLDL	mmol/l
Free cholesterol in chylomicrons and extremely large VLDL	mmol/l
Triglycerides in chylomicrons and extremely large VLDL	mmol/l
**Very large VLDL (average diameter 64 nm)**	
Concentration of very large VLDL particles	mmol/l
Total lipids in very large VLDL	mmol/l
Phospholipids in very large VLDL	mmol/l
Cholesterol in very large VLDL	mmol/l
Cholesteryl esters in very large VLDL	mmol/l
Free cholesterol in very large VLDL	mmol/l
Triglycerides in very large VLDL	mmol/l
**Large VLDL (average diameter 53.6 nm)**	
Concentration of large VLDL particles	mmol/l
Total lipids in large VLDL	mmol/l
Phospholipids in large VLDL	mmol/l
Cholesterol in large VLDL	mmol/l
Cholesteryl esters in large VLDL	mmol/l
Free cholesterol in large VLDL	mmol/l
Triglycerides in large VLDL	mmol/l

Metabolite	Unit
**Medium VLDL (average diameter 44.5 nm)**	
Concentration of medium VLDL particles	mmol/l
Total lipids in medium VLDL	mmol/l
Phospholipids in medium VLDL	mmol/l
Cholesterol in medium VLDL	mmol/l
Cholesteryl esters in medium VLDL	mmol/l
Free cholesterol in medium VLDL	mmol/l
Triglycerides in medium VLDL	mmol/l
**Small VLDL (average diameter 36.8 nm)**	
Concentration of small VLDL particles	mmol/l
Total lipids in small VLDL	mmol/l
Phospholipids in small VLDL	mmol/l
Cholesterol in small VLDL	mmol/l
Cholesteryl esters in small VLDL	mmol/l
Free cholesterol in small VLDL	mmol/l
Triglycerides in small VLDL	mmol/l
**Very small VLDL (average diameter 31.3 nm)**	
Concentration of very small VLDL particles	mmol/l
Total lipids in very small VLDL	mmol/l
Phospholipids in very small VLDL	mmol/l
Cholesterol in very small VLDL	mmol/l
Cholesteryl esters in very small VLDL	mmol/l
Free cholesterol in very small VLDL	mmol/l
Triglycerides in very small VLDL	mmol/l
**IDL (average diameter 28.6 nm)**	
Concentration of IDL particles	mmol/l
Total lipids in IDL	mmol/l
Phospholipids in IDL	mmol/l
Cholesterol in IDL	mmol/l
Cholesteryl esters in IDL	mmol/l
Free cholesterol in IDL	mmol/l
Triglycerides in IDL	mmol/l
**Large LDL (average diameter 25.5 nm)**	
Concentration of large LDL particles	mmol/l
Total lipids in large LDL	mmol/l
Phospholipids in large LDL	mmol/l
Cholesterol in large LDL	mmol/l
Cholesteryl esters in large LDL	mmol/l
Free cholesterol in large LDL	mmol/l
Triglycerides in large LDL	mmol/l
**Medium LDL (average diameter 23 nm)**	
Concentration of medium LDL particles	mmol/l
Total lipids in medium LDL	mmol/l
Phospholipids in medium LDL	mmol/l
Cholesterol in medium LDL	mmol/l
Cholesteryl esters in medium LDL	mmol/l
Free cholesterol in medium LDL	mmol/l
Triglycerides in medium LDL	mmol/l

All listed biomarkers are available for Serum and Heparin plasma samples.

*Biomarkers marked with * are not available for EDTA plasma samples.*
*Biomarkers marked with ** are not available for Citrate plasma samples.*

List of Biomarkers

Metabolite	Unit
**Small LDL (average diameter 18.7 nm)**	
Concentration of small LDL particles	mmol/l
Total lipids in small LDL	mmol/l
Phospholipids in small LDL	mmol/l
Cholesterol in small LDL	mmol/l
Cholesteryl esters in small LDL	mmol/l
Free cholesterol in small LDL	mmol/l
Triglycerides in small LDL	mmol/l
**Very large HDL (average diameter 14.3 nm)**	
Concentration of very large HDL particles	mmol/l
Total lipids in very large HDL	mmol/l
Phospholipids in very large HDL	mmol/l
Cholesterol in very large HDL	mmol/l
Cholesteryl esters in very large HDL	mmol/l
Free cholesterol in very large HDL	mmol/l
Triglycerides in very large HDL	mmol/l
**Large HDL (average diameter 12.1 nm)**	
Concentration of large HDL particles	mmol/l
Total lipids in large HDL	mmol/l
Phospholipids in large HDL	mmol/l
Cholesterol in large HDL	mmol/l
Cholesteryl esters in large HDL	mmol/l
Free cholesterol in large HDL	mmol/l
Triglycerides in large HDL	mmol/l
**Medium HDL (average diameter 10.9 nm)**	
Concentration of medium HDL particles	mmol/l
Total lipids in medium HDL	mmol/l
Phospholipids in medium HDL	mmol/l
Cholesterol in medium HDL	mmol/l
Cholesteryl esters in medium HDL	mmol/l
Free cholesterol in medium HDL	mmol/l
Triglycerides in medium HDL	mmol/l
**Small HDL (average diameter 8.7 nm)**	
Concentration of small HDL particles	mmol/l
Total lipids in small HDL	mmol/l
Phospholipids in small HDL	mmol/l
Cholesterol in small HDL	mmol/l
Cholesteryl esters in small HDL	mmol/l
Free cholesterol in small HDL	mmol/l
Triglycerides in small HDL	mmol/l
**Relative lipoprotein lipid concentrations**	
**Chylomicrons and extremely large VLDL ratios**	
Phospholipids to total lipids ratio in chylomicrons and extremely large VLDL	%
Cholesterol to total lipids ratio in chylomicrons and extremely large VLDL	%
Cholesteryl esters to total lipids ratio in chylomicrons and extremely large VLDL	%
Free cholesterol to total lipids ratio in chylomicrons and extremely large VLDL	%

Metabolite	Unit
Triglycerides to total lipids ratio in chylomicrons and extremely large VLDL	%
**Very large VLDL ratios**	
Phospholipids to total lipids ratio in very large VLDL	%
Cholesterol to total lipids ratio in very large VLDL	%
Cholesteryl esters to total lipids ratio in very large VLDL	%
Free cholesterol to total lipids ratio in very large VLDL	%
Triglycerides to total lipids ratio in very large VLDL	%
**Large VLDL ratios**	
Phospholipids to total lipids ratio in large VLDL	%
Cholesterol to total lipids ratio in large VLDL	%
Cholesteryl esters to total lipids ratio in large VLDL	%
Free cholesterol to total lipids ratio in large VLDL	%
Triglycerides to total lipids ratio in large VLDL	%
**Medium VLDL ratios**	
Phospholipids to total lipids ratio in medium VLDL	%
Cholesterol to total lipids ratio in medium VLDL	%
Cholesteryl esters to total lipids ratio in medium VLDL	%
Free cholesterol to total lipids ratio in medium VLDL	%
Triglycerides to total lipids ratio in medium VLDL	%
**Small VLDL ratios**	
Phospholipids to total lipids ratio in small VLDL	%
Cholesterol to total lipids ratio in small VLDL	%
Cholesteryl esters to total lipids ratio in small VLDL	%
Free cholesterol to total lipids ratio in small VLDL	%
Triglycerides to total lipids ratio in small VLDL	%
**Very small VLDL ratios**	
Phospholipids to total lipids ratio in very small VLDL	%
Cholesterol to total lipids ratio in very small VLDL	%
Cholesteryl esters to total lipids ratio in very small VLDL	%
Free cholesterol to total lipids ratio in very small VLDL	%
Triglycerides to total lipids ratio in very small VLDL	%
**IDL ratios**	
Phospholipids to total lipids ratio in IDL	%
Cholesterol to total lipids ratio in IDL	%
Cholesteryl esters to total lipids ratio in IDL	%
Free cholesterol to total lipids ratio in IDL	%
Triglycerides to total lipids ratio in IDL	%
**Large LDL ratios**	
Phospholipids to total lipids ratio in large LDL	%
Cholesterol to total lipids ratio in large LDL	%
Cholesteryl esters to total lipids ratio in large LDL	%
Free cholesterol to total lipids ratio in large LDL	%
Triglycerides to total lipids ratio in large LDL	%

Metabolite	Unit
**Medium LDL ratios**	
Phospholipids to total lipids ratio in medium LDL	%
Cholesterol to total lipids ratio in medium LDL	%
Cholesteryl esters to total lipids ratio in medium LDL	%
Free cholesterol to total lipids ratio in medium LDL	%
Triglycerides to total lipids ratio in medium LDL	%
**Small LDL ratios**	
Phospholipids to total lipids ratio in small LDL	%
Cholesterol to total lipids ratio in small LDL	%
Cholesteryl esters to total lipids ratio in small LDL	%
Free cholesterol to total lipids ratio in small LDL	%
Triglycerides to total lipids ratio in small LDL	%
**Very large HDL ratios**	
Phospholipids to total lipids ratio in very large HDL	%
Cholesterol to total lipids ratio in very large HDL	%
Cholesteryl esters to total lipids ratio in very large HDL	%
Free cholesterol to total lipids ratio in very large HDL	%
Triglycerides to total lipids ratio in very large HDL	%
**Large HDL ratios**	
Phospholipids to total lipids ratio in large HDL	%
Cholesterol to total lipids ratio in large HDL	%
Cholesteryl esters to total lipids ratio in large HDL	%
Free cholesterol to total lipids ratio in large HDL	%
Triglycerides to total lipids ratio in large HDL	%
**Medium HDL ratios**	
Phospholipids to total lipids ratio in medium HDL	%
Cholesterol to total lipids ratio in medium HDL	%
Cholesteryl esters to total lipids ratio in medium HDL	%
Free cholesterol to total lipids ratio in medium HDL	%
Triglycerides to total lipids ratio in medium HDL	%
**Small HDL ratios**	
Phospholipids to total lipids ratio in small HDL	%
Cholesterol to total lipids ratio in small HDL	%
Cholesteryl esters to total lipids ratio in small HDL	%
Free cholesterol to total lipids ratio in small HDL	%
Triglycerides to total lipids ratio in small HDL	%

All listed biomarkers are available for Serum and Heparin plasma samples.

*Biomarkers marked with * are not available for EDTA plasma samples.*
*Biomarkers marked with ** are not available for Citrate plasma samples.*

Nightingale

Nightingale Health Ltd. provides a NMR (Nuclear Magnetic Resonance) based metabolomics technology, supplying biomarker analysis services for human blood, urine, CSF and umbilical cord blood samples. By measuring biomarkers from multiple pathways in a single experiment, Nightingale equips public health researchers with comprehensive insights into the effects of lifestyle factors and future disease risk, accelerating future breakthroughs in precision medicine. In the long term, the company plans to fully integrate its services into clinical practice, helping to empower patients to follow-up on their own well-being and take proactive steps to stay healthy.

See also

Nightingale CSF Biomarker Analysis Service
Nightingale Urine Biomarker Analysis Service
Nightingale Cord Blood Biomarker Analysis Service

LB-0428 11/2019

Nightingale Urine Biomarker Analysis Service

Fully quantitative metabolic data for novel scientific discoveries powered by recent breakthrough in metabolomics technology

Powerful platform enabled by NMR

Robust and highly reproducible results

Our fully automated analysis process is constantly monitored. NMR technology allows high reproducibility which ensures consistent and reliable results across all sample sets.

Fast, cost-efficient and scalable technology

We use a high-throughput NMR technology which ensures efficient analysis for sample sets of all sizes without batch effects.

Accurate and fully quantified metabolic data

Not only our measurement of the samples, but also our quantification process of the NMR spectral data is fully automated, which provides precise and accurate metabolite results in absolute concentration units.

Comprehensive overview of an individual's health

Biomarkers in our panel provide a physiologically meaningful picture of the overall health making it possible to explore novel connections between metabolites and an individual's health status.

Tech specifications

**Technology/ method**	1H NMR Spectroscopy, Nightingale Health's proprietary analysis
**Sample volume**	500 µL
**Number of biomarkers**	54
**Result units**	Absolute biomarker quantification (mmol/l and ratio to creatinine)
**Required sample storage**	Long-term storage -70°C or below

Application examples

- Early risk detection and prognostics of type 1 and type 2 diabetes as well as diabetic complications, especially for diabetic kidney disease.
- Molecular understanding of cardiometabolic risk factors such as adiposity and body fat distribution, and what role they play in the disease etiology.
- Genetic regulation of urine metabolism and further implications to disease etiology
- Exploring metabolic effects of an individual's diet and lifestyle on health

List of Biomarkers

Available in March 2020

Metabolite	Unit
**Amino acids**	
Alanine	mmol/l & ratio to creatinine
Glutamine	mmol/l & ratio to creatinine
Glycine	mmol/l & ratio to creatinine
Histidine	mmol/l & ratio to creatinine
Taurine	mmol/l & ratio to creatinine
Threonine	mmol/l & ratio to creatinine
Tryptophan	mmol/l & ratio to creatinine
Branched-chain amino acids	
Isoleucine	mmol/l & ratio to creatinine
Leucine	mmol/l & ratio to creatinine
Valine	mmol/l & ratio to creatinine
Aromatic amino acids	
Tyrosine	mmol/l & ratio to creatinine
**Dietary metabolites**	
2-Furoylglycine	mmol/l & ratio to creatinine
3-Methylhistidine	mmol/l & ratio to creatinine
Arabinose	mmol/l & ratio to creatinine
Ethanol	mmol/l & ratio to creatinine
HPHPA	mmol/l & ratio to creatinine
Mannitol	mmol/l & ratio to creatinine
Proline betaine*	mmol/l & ratio to creatinine
Propylene glycol*	mmol/l & ratio to creatinine
Quinic acid	mmol/l & ratio to creatinine
Sucrose	mmol/l & ratio to creatinine
Trans-aconitate	mmol/l & ratio to creatinine
Xanthosine	mmol/l & ratio to creatinine
Xylose	mmol/l & ratio to creatinine

Metabolite	Unit
**Fluid balance**	
Creatinine	mmol/l
**Glycolysis related metabolites**	
cis-Aconitate	mmol/l & ratio to creatinine
Citrate	mmol/l & ratio to creatinine
Glucose	mmol/l & ratio to creatinine
Lactate	mmol/l & ratio to creatinine
**Ketone bodies**	
Acetate	mmol/l & ratio to creatinine
**Microbial metabolism**	
3-Hydroxyhippurate	mmol/l & ratio to creatinine
Dimethylamine	mmol/l & ratio to creatinine
Trimethylamine N-oxide	mmol/l & ratio to creatinine
**Miscellaneous**	
2-Hydroxyisobutyrate	mmol/l & ratio to creatinine
3-Hydroxyisobutyrate	mmol/l & ratio to creatinine
3-Hydroxyisovalerate	mmol/l & ratio to creatinine
4-Deoxyerythronic acid	mmol/l & ratio to creatinine
4-Deoxythreonate	mmol/l & ratio to creatinine
4-Hydroxyhippurate	mmol/l & ratio to creatinine
Allantoin	mmol/l & ratio to creatinine
Creatine	mmol/l & ratio to creatinine
Ethanolamine	mmol/l & ratio to creatinine
Formate	mmol/l & ratio to creatinine
Glycolate	mmol/l & ratio to creatinine
Hypoxanthine	mmol/l & ratio to creatinine
Indoxyl Sulfate	mmol/l & ratio to creatinine
Pseudouridine	mmol/l & ratio to creatinine
Pyroglutamate	mmol/l & ratio to creatinine
Urea	mmol/l & ratio to creatinine

Metabolite	Unit
**Nicotinate and nicotinamide metabolism**	
1-Methylnicotinamide	mmol/l & ratio to creatinine
Trigonelline	mmol/l & ratio to creatinine
**Phenylalanine metabolism**	
Hippurate	mmol/l & ratio to creatinine
**Pyrimidine metabolism**	
3-Aminoisobutyrate	mmol/l & ratio to creatinine
Uracil	mmol/l & ratio to creatinine

** Preliminary biomarkers*

Nightingale

Nightingale Health Ltd. provides a NMR (Nuclear Magnetic Resonance) based metabolomics technology, supplying biomarker analysis services for human blood, urine, CSF and umbilical cord blood samples. By measuring biomarkers from multiple pathways in a single experiment, Nightingale equips public health researchers with comprehensive insights into the effects of lifestyle factors and future disease risk, accelerating future breakthroughs in precision medicine. In the long term, the company plans to fully integrate its services into clinical practice, helping to empower patients to follow-up on their own well-being and take proactive steps to stay healthy.

See also

Nightingale CSF Biomarker Analysis Service
Nightingale Blood Analysis Service
Nightingale Cord Blood Biomarker Analysis Service
