# Supplementary material for: Cohort profile: The Belgian I AM frontier prospective cohort study for comprehensive health outcome exploration
Source: PLoS One. 2025 Jun 12;20(6):e0326024. doi: 10.1371/journal.pone.0326024 (PMC12161581; doi:10.1371/journal.pone.0326024)
Supplement: S1 File — (PDF) [file pone.0326024.s008.pdf]

Document 1: Overview Intake, Weekly, and Monthly questionnaire

Table 1: Overview Intake, Weekly, and Monthly questionnaire

Questions	*Intake*	*Weekly*	*Monthly*
Date of birth *	x		
Mobile number			x
National register number			x
General practitioner			x
Where do you live most of the time at the moment?	x		x
Postal code?	x		
Country?	x		
How many years have you lived at your current address?	x		
Including yourself, how many people live together in your household? (Include those who usually live in the house, such as students who live away from home, partners in Defence or professions such as pilots, ...)*	x		
What is your relationship with the people in the household?	x		
Which of the following describes your current work situation?*	x		
How many kilometres is the distance between your home and your work?*	x		
How much time do you spend on average travelling between your home and your work, one way?*	x		
How many times per week do you make this journey from your home to your work (e.g. if you drive from home to VITO and back 5 days, then answer 5, not 10)?*	x		
In a typical week, how many hours do you spend at work?*	x		
In a typical week, how many hours do you spend at home working?*	x		
What type of transport do you use to get to and from work?	x		
Do you usually walk or stand up at work?*	x		
Does your work involve heavy manual or physical work?*	x		
What is the highest level of education or vocational training you have completed?*	x		
What is your genetic background? (take into account the origin of both father and mother)	x		
On how many days during the past 7 days did you do activities that require moderate physical exertion such as carrying light loads, cycling at a normal pace, light sports such as doubles in tennis, swimming, etc.? Please note that walking or walking are NOT included in these activities.*	x		x
How much time do you usually spend on such a day doing moderate physical activities?*	x		x
On how many days during the past 7 days did you do activities that require heavy physical exertion such as heavy lifting, digging, aerobics, jogging, football, etc.?*	x		x
How much time do you usually spend on such a day doing heavy physical activities?*	x		x
On how many days during the past 7 days did you walk or walk for at least 10 minutes at a time?*	x		x
On how much time do you usually spend walking or walking on such a day?*	x		x
At what pace do you usually walk or walk? Is this ...*	x		x
Do you exercise for at least half an hour every day?*	x		x
How would you best describe your leisure activities in the past 4 weeks?	x		x
Have you done any of the following in the past 4 weeks?	x		x
How often have you done light household chores (e.g. pruning, lawn, watering, vacuuming, dusting, etc.) in the past 4 weeks?*	x		x

How often have you done heavy sports (e.g. running, etc.) in the past 4 weeks?*	x		x
How often have you walked for pleasure in the past 4 weeks?*	x		x
How often have you done other physical activities in the past 4 weeks, such as swimming, cycling, etc.?*	x		x
How many times a day do you take the stairs? (Approximately 10 steps per flight of stairs)*	x		x
How many hours do you spend driving on an average day? *	x		
Which means of transport have you used most in the past 4 weeks? Please note that this does not apply to your journey to work.	x		x
How many hours do you spend on an average day using your computer, or mobile devices such as your phone, tablet, etc.? (Not at work)*	x		
How many hours do you spend on an average day watching TV?*	x		
How many hours of sleep do you get in 24 hours? (including any naps)*	x		
On an average day, how easy is it for you to get up in the morning?*	x		
Do you have trouble falling asleep at night or do you wake up in the middle of the night?*	x		
Does your partner or a close family member or friend complain about your snoring?*	x		
How likely is it that you fall asleep during the day or fall asleep when you don't want to?*	x		
Have you smoked in the past?*	x		
Do you smoke now?*	x		
Does anyone in your household smoke?*	x		
Do you regularly take any of the following supplements?	x		
How often do you eat vegetables or salad, excluding juice and potatoes?*	x		
How often do you eat fruit, excluding juice? *	x		
Have you ever taken medication for high blood pressure?*	x		
Have you ever been diagnosed with high blood sugar levels? Also consider a hospital stay, routine check-up or gestational diabetes*	x		
Do you have family members with diabetes (type 1 or type 2)?*	x		
What did you eat and drink yesterday; always state everything you ate and drank (we only ask for water at the bottom). If you do not know the weight of what you ate, please fill in as accurate a description as possible of the quantity (e.g. number of slices, pieces, etc.).	x		x
Briefly describe whether and what (heavy) physical exertion you have done in the past 24 hours:	x		x
Have you smoked in the past 24 hours?	x		x
How many hours in the past 24 hours have you been in a room where smoking was taking place (by yourself or others)? *	x		x
Have you taken any of the following supplements in the past 24 hours?	x		x
Have you taken any medication in the past 24 hours? *	x		x
Sleep problems (not being able to fall asleep, waking up in the middle of the night)	x	x	
Weight change (gained or lost 2 kg)	x	x	
Back pain	x	x	
Constipation	x	x	
Dizziness	x	x	
Diarrhea	x	x	
Feeling uneasy	x	x	
Constant fatigue	x	x	
Headache	x	x	

Migraine headache	x	x
Nausea and/or vomiting	x	x
Heartburn or indigestion	x	x
Stomach pain (e.g. cramps)	x	x
Hot or cold shivers	x	x
Shaking hands	x	x
Thumping or racing heart	x	x
Loss appetite	x	x
Shortness of breath when not doing physical activity	x	x
Numbness or tingling in parts of your body	x	x
Feeling faint	x	x
Heart or chest pain	x	x
Low energy levels	x	x
Blocked nose or head	x	x
Blurred vision	x	x
Muscle tension or pain	x	x
Muscle cramps	x	x
Severe aches and pains	x	x
Acne	x	x
Bruises	x	x
Nose bleeds	x	x
Tense muscles	x	x
Tight ligaments	x	x
Cold or cough	x	x
General feeling in the past week:*	x	x
Have you had a hair sample taken?*	x	
When did you last wash your hair?	x	
Did you use care products such as gel, oil, hair cream, conditioner after the last wash?*	x	
Have you colored your hair in the last 6 months (color rinse, highlights, bleaching, etc.)?*	x	
Have you changed the structure of your hair in the last 6 months (perm, straightening, etc.)?*	x	
