# Supplementary material for: Cohort profile: The Belgian I AM frontier prospective cohort study for comprehensive health outcome exploration
Source: PLoS One. 2025 Jun 12;20(6):e0326024. doi: 10.1371/journal.pone.0326024 (PMC12161581; doi:10.1371/journal.pone.0326024)
Supplement: S2 File — (TIF) [file pone.0326024.s009.pdf]

Document 2: Overview WGS questionnaire

Questions	*Pre-Results*	*Post-Results*
I am curious about my genetic predisposition to diseases.	x	
Would you like to receive all your raw genome results? Raw results are the genome data without editing or other manipulation.	x	
Would you like to be informed about a 1 in 100 risk (i.e. 1% chance) of a serious preventable condition?	x	
Would you like to receive information that is uncertain and cannot be interpreted at this time?	x	x
Would you like to receive information about life-threatening conditions that cannot be prevented?	x	x
Would you like to receive information about life-threatening conditions that can be prevented?	x	x
Would you like to receive information about conditions that are serious (but not life-threatening) and that cannot be prevented?	x	x
Would you like to receive information that shows how you might respond to different treatments or medications (e.g. cholesterol-lowering drugs, antidepressants, etc.)?	x	
Would you like to receive information that tells you whether you are a carrier of a condition that could be relevant to your children?	x	
Would you like to receive information that is not immediately relevant but could be useful later in life (e.g. regarding very late onset cancer or predisposition to stroke)?	x	
Would you like to receive information that is unlikely to pose a serious health risk (e.g. mild vision problems)?	x	
How satisfied are you with your choice to have your DNA analyzed?		x
As a result of the genome analysis, I feel that I could adjust my decisions and behavior to benefit my health.		x
I was able to understand new information and its meaning based on the information letter I received.		x
I am disappointed that the results of the genome analysis did not provide me with more information.		x
Have you shared your genome results with blood relatives? (children, siblings, parents)		x
Have you shared your genome results with non-blood relatives? (e.g. partner, friends, ...)		x
Are you considering genetic counseling (consultation with a genetics specialist) after receiving your results or have you already used it?		x
Do you think that people who have received their individual genome analysis results need genetic counseling?		x
Which of the following options would you consult after receiving your genome results or have you already consulted?		x
Do you think that the GP should help people who have a genome test to interpret the results?		x
Would you recommend others to have a genome test?		x
I think that everyone should have a genome test so that timely intervention can be done in the event of illness.		x
I think that genetic information should be a standard part of my medical file.		x
If we were to repeat the I AM frontier study, which results would we definitely have to provide to participants again?		x
Considering all the results you received from us, which did you find most valuable?		x
Were there any aspects that you felt were missing from the study?		x

The information I received during the I AM frontier period has influenced how I have maintained my health since then.	x
Have you made a lifestyle change - something you did, started or stopped doing as a result of your personal results?	x
